# The Resistance-Nodulation-Division efflux pump EefABC is highly conserved within lineages of E. coli commonly associated with infection

**DOI:** 10.1099/mgen.0.001593

**Published:** 2026-01-12

**Authors:** Hannah L. Pugh, Elizabeth M. Darby, Leah Burgess, Abigail L. Colclough, Asti-Rochelle Meosa John, Steven Dunn, Christopher Connor, Eoughin A. Perry, Alan McNally, Vassiliy N. Bavro, Jessica M. A. Blair

**Affiliations:** 1 Department of Microbes, Infection and Microbiomes, School of Infection, Inflammation and Immunology, College of Medicine and Health, Institute of Microbiology and Infection, University of Birmingham, Edgbaston, Birmingham, B15 2TT, UK; 2 The Francis Crick Institute, 1 Midland Road, London, NW1 1AT, UK; 3 Department of Microbiology and Immunology at the Peter Doherty Institute for Infection and Immunity, University of Melbourne, Melbourne, Victoria, 3010, Australia; 4 School of Life Sciences, University of Essex, Colchester, CO4 3SQ, UK

**Keywords:** antibiotic resistance, antimicrobial resistance, *E. coli*, efflux pump

## Abstract

Resistance-nodulation-division (RND) efflux pumps confer multidrug resistance in Gram-negative bacteria and are critical for many physiological functions including virulence and biofilm formation. The common *Escherichia coli* laboratory strain, K-12 MG1655, has six recognized RND transporters (AcrB, AcrD, AcrF, CusA, MdtBC and MdtF). However, by studying >20,000 *E*. *coli* assemblies, we show that *E. coli* belonging to phylogroups B2, D, E, F and G, which are commonly associated with infection, possess an additional, seventh RND transporter, EefB. It is found in a five-gene operon, *eefRABCD*, which also encodes a TetR family transcription factor, a periplasmic adapter protein, an outer membrane factor and major facilitator superfamily pump. In contrast, *E. coli* from phylogroups A, B1 and C, generally containing environmental/commensal strains, do not encode the operon. Where the *eefRABCD* operon is present, it was highly conserved. In fact, conservation levels were comparable to that of the major RND efflux system AcrAB-TolC, suggesting an important biological function. Protein modelling shows that this pump is distinct from endogenous *E. coli* RND systems with unique structural features. However, unlike other RND efflux systems, EefABC does not appear to transport antimicrobials and instead may be important for infection or survival in the host environment.

**Impact Statement**

Efflux pumps are molecular machines that export molecules out of bacterial cells [113]. The efflux pumps belonging to the resistance-nodulation-division (RND) family are particularly important as they export antibiotics out of Gram-negative bacterial cells, contributing to antibiotic resistance. The important human pathogen, *Escherichia coli*, has been previously reported to have six RND pumps. However, we show that phylogroups of *E. coli* commonly associated with infection encode a seventh RND pump, EefABC, which is highly conserved, suggesting an important biological function. While the function of EefABC in *E. coli* remains to be resolved, it does not seem to transport antimicrobial compounds. These findings are important because they reveal a new RND pump, potentially involved in virulence and survival in the host, which could represent a new therapeutic target. Additionally, it again shows that laboratory type strains of common bacterial pathogens are not representative of those that are infection causing.

## Data Summary

All genomic data used in this study were downloaded from open-access sources. Assembly barcodes of *Escherichia coli* assemblies downloaded from EnteroBase (https://enterobase.warwick.ac.uk/species/index/ecoli) are available at 10.6084/m9.figshare.29959187 [1]. NCBI and UniProt accession codes are provided in both the main text and supplementary information.

## Introduction

*Escherichia coli* is a leading cause of invasive bacterial infections in humans, causing a range of diseases from urinary tract infections to haemorrhagic shock [2]. However, *E. coli* is also found in the wider environment and is a common commensal, colonizing the gastrointestinal tract of both humans and animals. *E. coli* is a genetically diverse species that is divided into phylogroups (A, B1, B2, C, D, E, F and G) which are determined by genetic similarity [3]. Though virulence is not limited to specific phylogroups [4,5], extra-intestinal infection is most commonly associated with phylogroups B2 and D [3], while commensal or environmental lifestyles are mainly associated with phylogroups A and B1 [6]. In addition to phylogroups, *E. coli* isolates are also classified by sequence types (STs), determined by multi-locus sequence typing [7]. Pandemic clonal STs such as ST131 are responsible for high incidence rates of extra-intestinal *E. coli* infections and the carriage of genes conferring multidrug resistance [8,9].

Gram-negative bacteria, such as *E. coli*, possess efflux pumps of the resistance-nodulation-division (RND) family which are best known for the export of antimicrobials and biocides [10,13] with overexpression of these pumps conferring multidrug resistance (MDR) [14,15]. RND pumps form tripartite complexes with periplasmic adaptor proteins (PAPs) and outer membrane factor (OMF) to span the inner membrane, periplasmic space and the outer membrane [10]. In addition to MDR, RND pumps have been implicated in a wide range of additional functions, including virulence [16,18], biofilm production [19], decreased susceptibility to dyes, bile salts and fatty acids [12,20], export of polyamines and quorum sensing molecules [21], export of bacterial metabolites [22], copper and ion homeostasis [23,24] and motility [25,26].

Many Gram-negative bacteria encode multiple RND efflux pumps, with differing substrate specificities which are often expressed only under specific environmental conditions. However, the number of RND pumps is highly variable between bacterial lineages. The human-restricted pathogen *Neisseria gonorrhoeae* possesses only a single RND pump (MtrCDE), while bacterial species that can colonize multiple habitats generally possess more. *Salmonella enterica* serovar Typhimurium has five RND pumps, whereas *E. coli* is generally reported to have six: AcrB, AcrD, AcrF, MdtBC, MdtF and CusA [12]. The number of RND pumps can also vary within a genus. We have recently shown that species across the *Acinetobacter* genus possess between two and nine RND pumps, with species most commonly associated with human infection tending to encode more RND efflux pumps [27]. However this is not always the case, for example a study by Ma *et al*. found isolates of *N. gonorrhoeae* with loss of MtrC function were over-represented in the cervical environment [28]. We have also demonstrated that AcrF in the pathogenic *E. coli* O157:H7 is non-functional due to a conserved insertion containing two stop codons [29], showing that within a species not all efflux pumps present are always functional.

The expression of RND efflux pumps is controlled by a complex network of positive and negative regulators including repressors of the TetR family. We previously reported the presence of the TetR family regulator EefR in four of ten *E. coli* strains studied [30]. The *eefR* gene was encoded alongside genes annotated as *eefA* and *eefB* that are predicted to encode a PAP and RND pump, respectively. This RND pump was first reported in *Enterobacter aerogenes* (now reclassified as *Klebsiella aerogenes*), where *eefA, eefB* and *eefC* were found in a three-gene operon without the regulator, *eefR* [31]. In that study, the *eefABC* operon was not expressed under laboratory conditions due to transcriptional silencing by H-NS [31]; however, overexpression was found to confer resistance to erythromycin [32]. The EefABC RND pump has also been reported in *Klebsiella pneumoniae,* where it has been linked to virulence, as deletion of *eefA* was found to reduce both colonization of the gastrointestinal tract and tolerance to low pH [33].

In *E. coli*, the *eefABC* operon is absent from the widely studied strain K-12 but has been reported in a single highly drug-resistant environmental isolate*, E. coli* SMS-3-5. In this isolate, the efflux pump was part of a larger gene cluster which included the regulator *eefR* and a major facilitator superfamily (MFS) efflux pump *eefD* [34]. To date, the wider prevalence of the *eefRABCD* operon across the diversity of the *E. coli* population and its function in *E. coli* are both still unknown. Here, we demonstrate that (1) EefABC is exclusively found in clinically relevant phylogroups of *E. coli* and is highly conserved, (2) homology modelling reveals that the pump has several distinctive structural features compared to other RND efflux pumps and (3) neither EefABC nor EefD transport clinically relevant antimicrobials but can transport dyes.

## Methods

### Prevalence and conservation of *eefRABCD* across *E. coli* and related species

The *eefB* gene from *E. coli* SMS-3-5 (CP000970.1) was aligned to *E. coli* genomes (taxid 562) within the NCBI RefSeq Genome Database.

A total of 20,013 *E. coli* genome assemblies from 38 STs (Achtman scheme) [35] were downloaded from EnteroBase [36] for the determination of *eef* conservation across *E. coli*. Duplicates were removed based upon MASH (v 2.2.2) distances [37]. A custom blast database (blast v 2.10.0) [38] was generated from the sequences of *eefR, eefA, eefB, eefC* and *eefD* of *E. coli* ATCC 25922 (CP009072.1) and used with ABRicate (v 0.9.8) [39] to identify the presence and conservation of *eef* genes across the *E. coli* assemblies. Where an *eef* gene was found to be split across multiple contigs within an assembly, the assembly was removed from analysis as it was not possible to confirm from the assemblies alone whether this was due to a sequencing or assembly error, or the interruption of the gene.

The *eefRABCD* gene sequences from *E. coli* ATCC 25922 were also used to determine whether *Shigella* species encode the operon. Assemblies of *Shigella boydii* (*n*=495), *Shigella dysenteriae* (*n*=497), *Shigella flexneri* (*n*=499) and *Shigella sonnei* (*n*=500) were downloaded from EnteroBase. As with *E. coli,* duplicates were identified using MASH and removed prior to running ABRicate. Assemblies containing *eef* genes split over multiple contigs were removed from the analysis as with *E. coli*. The *eefRABCD* genes from *E. coli* ATCC 25922 were also aligned to *Salmonella* (taxid 590), *Photorhabdus* (taxid 29487), *Yersinia* (taxid 629), *Serratia* (taxid 613), *Pseudomonas* (taxid 286), *Enterobacter* (taxid 547) and *Acinetobacter* (taxid 469) genomes to identify any homologues in related Gammaproteobacteria; however, this was achieved using the NCBI RefSeq Genome Database.

### The phylogenetic context of *eefRABCD*

Five assemblies of each *E. coli* ST (excluding ST84 where *n=*2) and *Escherichia fergusonii* were chosen at random to generate a phylogenetic tree. Assemblies were annotated using Prokka (v 1.14.6) [40] with subsequent GFF files used as input for Roary (v 3.13.0) [41]. The core gene alignment produced by Roary was used to construct a GTR-gamma tree with 100 bootstraps using RaXmL (v 8.2.12) [42]. Trees were visualized and annotated using iTOL [43].

### Genomic context of *eefRABCD*

Due to limited availability of RefSeq genomes for some *E. coli* STs used in this work, seven were chosen at random. Reference sequences were downloaded from NCBI NC_004431.1 (ST73), NC_007946.1 (ST95), NZ_HG941718.1 (ST131), NC_002695.2 (ST11), NC_000913.3 (ST10), NC_011751.1 (ST69) and NZ_CP035350.1 (ST617). The location of the *eef* operon was identified in the ST73, ST95, ST131 and ST11 and downloaded along with the flanking 10,000 bp. The homologous regions in ST10, ST69 and ST617 were also identified and downloaded. Alignments of the genomic regions in all seven reference sequences were performed using EasyFig (v 2.2.2) [44].

Genomes of additional bacterial species, *Escherichia albertii* (CP070290.2), *Escherichia marmotae* (CP056165.1)*, K. aerogenes* (NZ_CP041925.1)*, K. pneumoniae* (NC_016845.1), *Enterobacter vonholyi* (VTUC01000001.1), *Enterobacter dykesii* (VTTY01000003.1), *Enterobacter wuhouensis* (SJOO01000006.1), *Enterobacter kobei* (KI973153.1), *Enterobacter chengduensis* (CP043318.1), *S. boydii* (CP026836.1)*, S. dysenteriae* (CP026774.1), *S. flexneri* (AE005674.2) and *S. sonnei* (CP055292.1) were downloaded from the NCBI. The visualization of the genomic context of the *eef* operon and homologues was achieved using EasyFig. Mapping of insertion sequences in *S. dysenteriae* was done using ISEScan (v 1.7.2.3) [45].

### Homology modelling and structural analysis

Multiple sequence alignments (MSAs) were prepared using MAFFT and NJ/UPGMA phylogeny algorithms as implemented in MAFFT v 7 server (https://mafft.cbrc.jp/) [46]. Phylo.io was used for phylogenetic guide tree visualization [47]. Structural annotations of the MSA sequences were done with Espript 3 (http://espript.ibcp.fr) [48].

For homology modelling, I-TASSER [49] was used in manual mode with assignment of templates and structural alignment, supplemented by SWISS-MODEL [50]. The following structural templates have been used for the specific protein modelling. EefA modelling: MexA (UniProt P52477) 2V4D.pdb [51] and AcrA (UniProt P0AE06) 5V5S.pdb [52]. AcrA was used as a template due to the smaller gaps in the alignment and better quality of available full-length template. EefB modelling: MexB (UniProt P52002) 3W9I.pdb [53] and AcrB (UniProt P31224) 2GIF.pdb [54]. EefC modelling: TolC (UniProt P02930) 1EK9.pdb [55], OprM (UniProt Q51487) 4Y1K.pdb [56], OprJ (UniProt Q51397) 5AZS.pdb [57], OprN (UniProt Q9I0Y7) 5AZO.pdb, 5AZP.pdb [57] and 5IUY.pdb [58]. EefD modelling: EmrD (UniProt P31442) 2GFP.pdb [59] and MdfA (UniProt P0AEY8) 4ZP0.pdb [60].

For the models of the protein oligomers and the complete EefABC tripartite pump, rigid-body structural docking of the homology models was used guided by the available cryogenic-electron microscopy (Cryo-EM) structure (5O66.pdb [52]), the results of which were cross-validated manually and obvious steric clashes removed using Coot [61]. Additional structural analysis and visualization were performed with PyMOL (PyMOL Molecular Graphics System, v 1.71 Schrödinger, LLC).

### Strains, plasmids and culture conditions

All strains used in this work are listed in Table S1 (available in the online Supplementary Material). Strains were grown in lysogeny broth (Merck) at 37 ˚C with aeration unless stated otherwise.

### Cloning of *eefABCD*

The *eefABC* operon was amplified from the chromosome of *E. coli* ATCC 25922 (NCTC 12241) using Q5 polymerase (New England Biolabs) and primers which incorporated the *Nde*I and *Xho*I restriction sites (Table S2). The amplicon was cloned into both pET21a (ampicillin resistant) and pET24a (kanamycin resistant) plasmids (Invitrogen) which are identical aside from their resistance cassette. No IPTG induction was used in this work.

The *eefD* gene was amplified from the *E. coli* ATCC 25922 chromosome using Q5 (New England Biolabs) and primers that incorporated the *ApaLI* and *PstI* restriction sites (Table S2). The amplicon was then cloned into pACYC177 (ATCC).

### Deletion of *eefABC* and *eefD* in *E. coli* ATCC 25922

Deletion of the genes encoding the RND system of the *eefRABCD* operon was achieved by homologous recombination [62]. However, due to the size of the *eefABC* operon, first *eefB* was interrupted, followed by the remaining *eefA* and *eefC* genes. An *eefD* knockout was generated independently using the same method. All primers are listed in Table S2.

### Determination of minimum inhibition concentration of antimicrobials, metals and dyes

Bacterial susceptibility to a range of antimicrobials and dyes was determined using the agar doubling dilution method described by the Clinical and Laboratory Standards Institute [63]. *E. coli* ATCC 25922 was used as a control to confirm antimicrobial efficacy in line with EUCAST guidelines [64]. For the susceptibility to metals, bile salts and polyamines, a broth microdilution method was used [63].

### Accumulation and efflux of ethidium bromide

Ethidium bromide (EtBr) accumulation was measured as previously described [65]. Briefly, EtBr was added to cells and the increase in fluorescence was measured over time.

Efflux activity was also assessed as previously described [65]. Here, cells were incubated in the presence of EtBr and carbonyl cyanine m-chlorophenyl hydrazone (CCCP) until fluorescence saturation was reached. Re-energization was achieved with glucose, and the rate of reduction in fluorescence was measured.

## Results

### The *eefRABCD* operon is present in *E. coli* phylogroups associated with infection

To first understand how widespread the *eefRABCD* operon is across *E. coli*, the NCBI RefSeq Genome Database was utilized. The *eefB* gene from *E. coli* SMS-3-5 was aligned to genomes belonging to *E. coli* (taxid 562) using NCBI nucleotide blast. The top 100 matches in the *eefB* alignment had 100% sequence coverage and ≥97.7% sequence identity (data not shown), suggesting that the *eefB* gene was present more widely across *E. coli*. One of the strains found to possess *eefB* was the well-characterized strain ATCC 25922. The *eef* operon in both *E. coli* SMS-3-5 and ATCC 25922 was found to be very similar, and as a result, the operon from ATCC 25922 was used as the reference in future work.

**Fig. 1.**
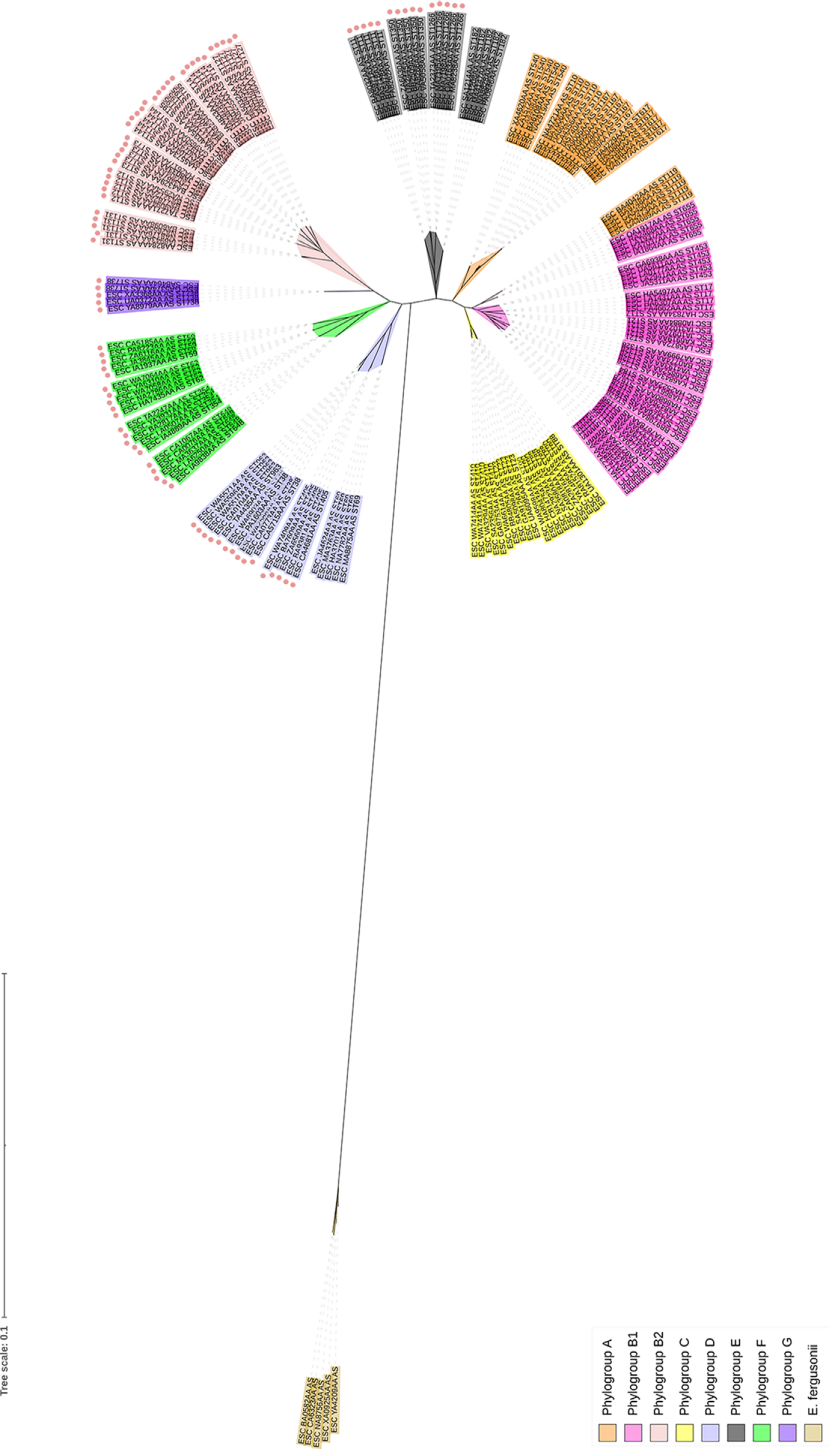
Phylogenetic structure of the assemblies used in this analysis and the distribution of *eefRABCD*. The tree was created using five assemblies per ST (exception ST84 *n=2*). Assemblies were chosen randomly. The tree was rooted using five *E. fergusonii* assemblies. The leaves are annotated with the ST group of the assembly and colour coded by phylogroup, and colours used to highlight phylogroups are shown in the legend on the bottom left corner. STs positive for the *eefRABCD* operon are marked with ●. A tree was generated using RaXmL and visualized on iTOL.

Next, we looked at the distribution of *eefRABCD* across *E. coli*. and Twenty thousand and thirteen assemblies were downloaded from the EnteroBase database representing 38 STs. Using MASH distances, 766 assemblies were identified as duplicates and removed from the data set. The presence of *eefR*, *eefA*, *eefB*, *eefC* and *eefD* in the remaining 19,247 assemblies was determined using ABRicate (Table S3). Interestingly, there was a clear divide between phylogroups where the *eefRABCD* operon was identified and those where it was completely absent (Fig. 1). The operon was not found in ST groups belonging to phylogroups A, B1 and C, which are more traditionally classified as environmental isolates. However, the *eefRABCD* operon was present in all ST groups of phylogroups B2, F and G, which are strongly associated with human infection and MDR. Notably, a single ST within phylogroups D and E lacked the operon, ST69 and ST182, respectively. Despite these two exceptions, a distinct divide between the A-B1-C and B2-D-E-F-G clades was identified, implying a clear evolutionary relationship.

### The *eefRABCD* operon is highly conserved across phylogroups B2, D, E, F and G

In *E. coli* assemblies positive for one *eefRABCD* gene, the entire operon was always present with gene sequences highly conserved. Using *E. coli* ATCC 25922 as a reference, gene coverage for each component of the *eef* operon was always greater than 98% (Table S4). The nucleotide percentage identity varied between genes and across phylogroups, though despite subtle variations, *eefR*, *eefA*, *eefB* and *eefD* averaged >99% overall. Interestingly, *eefC*, the gene coding for the OMF, was marginally more variable than the four other genes within the operon (Table 1).

**Table 1.** Conservation of *acrAB-tolC* and *eefRABCD* across *E. coli* phylogroups

Phylogroup	ST group	Mean nucleotide identity to *E. coli* ATCC 25922 (%)							
		*eefR*	*eefA*	*eefB*	*eefC*	*eefD*	*acrA*	*acrB*	*tolC*
**B2**	ST12	99.82	99.98	99.68	99.48	99.40	99.83	98.70	98.95
	ST127	99.12	99.64	99.61	99.42	99.48	99.75	99.46	98.57
	ST131	99.65	99.54	99.58	99.13	99.48	99.75	99.49	98.83
	ST14	98.91	98.49	99.41	99.12	99.57	99.83	99.70	98.83
	ST144	99.65	99.91	99.74	99.70	99.74	99.83	99.71	98.79
	ST73	100.00	100.00	100.00	100.00	99.97	100.00	100.00	99.93
	ST95	99.82	99.91	99.77	99.45	99.48	99.83	98.73	99.05
**D**	ST38	99.12	98.65	98.42	97.39	98.96	99.25	98.69	98.43
	ST405	99.45	98.32	99.00	99.20	99.14	99.33	98.67	98.05
	ST69	–	–	–	–	–	99.16	98.76	98.29
	ST963	99.12	98.66	98.42	97.38	98.97	99.25	98.70	98.65
**E**	ST11	99.12	97.95	98.06	97.53	98.37	99.33	98.53	98.52
	ST182	–	–	–	–	–	99.17	98.64	98.38
	ST350	99.47	98.03	98.38	97.62	98.25	99.08	98.70	98.44
	ST1266	99.47	98.22	98.46	97.57	98.19	99.08	98.76	98.65
**F**	ST354	98.93	98.84	99.52	97.07	99.66	99.08	98.97	98.37
	ST59	98.93	99.11	99.10	99.01	99.48	99.05	98.78	98.43
	ST62	99.12	98.75	99.39	98.76	99.23	99.15	98.79	98.10
	ST648	99.65	99.47	99.03	98.91	99.47	99.00	98.73	97.53
**G**	ST738	99.65	98.84	98.91	99.34	99.83	98.86	99.14	98.52
**Average gene conservation**		99.39	99.02	99.14	98.67	99.26	99.38	98.98	98.56

The extent of gene conservation for *acrA*,* acrB*,* tolC*,* eefR*,* eefA*,* eefB*,* eefC* and *eefD* across phylogroups B2, D, E, F, and G are highlighted as follows: ≥96% 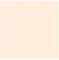, ≥97% 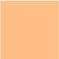, ≥98% ≥99% 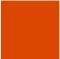.

To contextualize the extent of *eefRABCD* conservation, the conservation of *acrA*, *acrB* and *tolC*, which encode the critically important RND efflux pump AcrAB-TolC in *E. coli*, was determined. In general, the nucleoti de identity of the *eef* operon was conserved to a similar level as *acrAB-tolC*, all genes (excluding *eefC* in ST354) were >97% identical to those in *E. coli* ATCC 25922 (Table 1). However, conservation of both the *acrAB-tolC* and *eefABC* RND systems varied slightly at both the ST and phylogroup level. This suggests a strong selection pressure on the operon, indicating an important biological function.

Phylogroup B2 contains many clinically significant *E. coli* STs including the MDR ST131. Across phylogroup B2, *eefB* was generally more conserved than *acrB* with the *eef* genes having higher homology to the reference than the *acrAB-tolC* genes. However, in phylogroups D, E and F, the opposite was seen as the *acrAB-tolC* genes were more conserved than the *eefRABCD* genes. Though only one phylogroup G ST was used in the analysis, in ST738 *eefR, eefC* and *eefD* had the highest homology to the reference, with *acrA*, *acrB*, *eefA* and *eefB* all equally conserved.

### Phylogroups A, B1 and C have highly conserved *sapF-fabI* intergenic region in place of *eefRABCD*

**Fig. 2.**
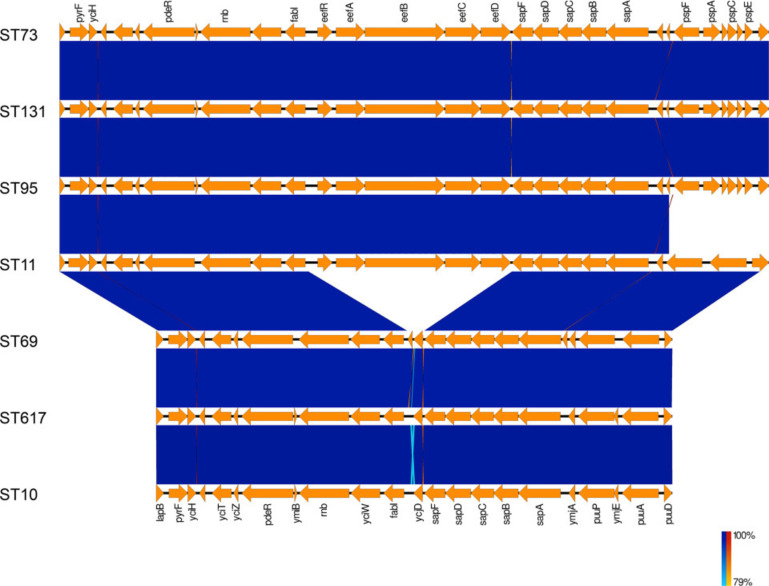
The genomic region of strains with and without *eefRABCD*. EasyFig alignment of the *eef* region in ST73 (B2), ST131 (B2), ST95 (B2), ST11 (E), ST69 (D), ST617 (A) and ST10 (A). The *eefRABCD* operon was consistently identified between *fabI* and *sapF*.

While *eefRABCD* was found to be highly conserved across phylogroups B2, D, E, F and G, it was completely absent from phylogroups A, B1 and C. To explore this further, RefSeq genomes of strains with and without the operon were downloaded from the NCBI and aligned using EasyFig (Fig. 2). In assemblies encoding the *eef* operon, it was always found at the same genomic location, that is, between the essential gene *fabI* and the non-essential gene *sapF*. FabI is an enoyl-[acyl-carrier-protein] reductase that is involved in fatty acid production [66], while SapF is a putrescine export protein belonging to the SapBCDF system [67]. Interestingly, in assemblies where the *eefRABCD* operon was absent, a hypothetical gene, *ycjD*, was annotated as present in the same genomic location. The *ycjD* ORF was 354 nt long and ran in the opposite orientation to *eefRABCD*.

**Fig. 3.**
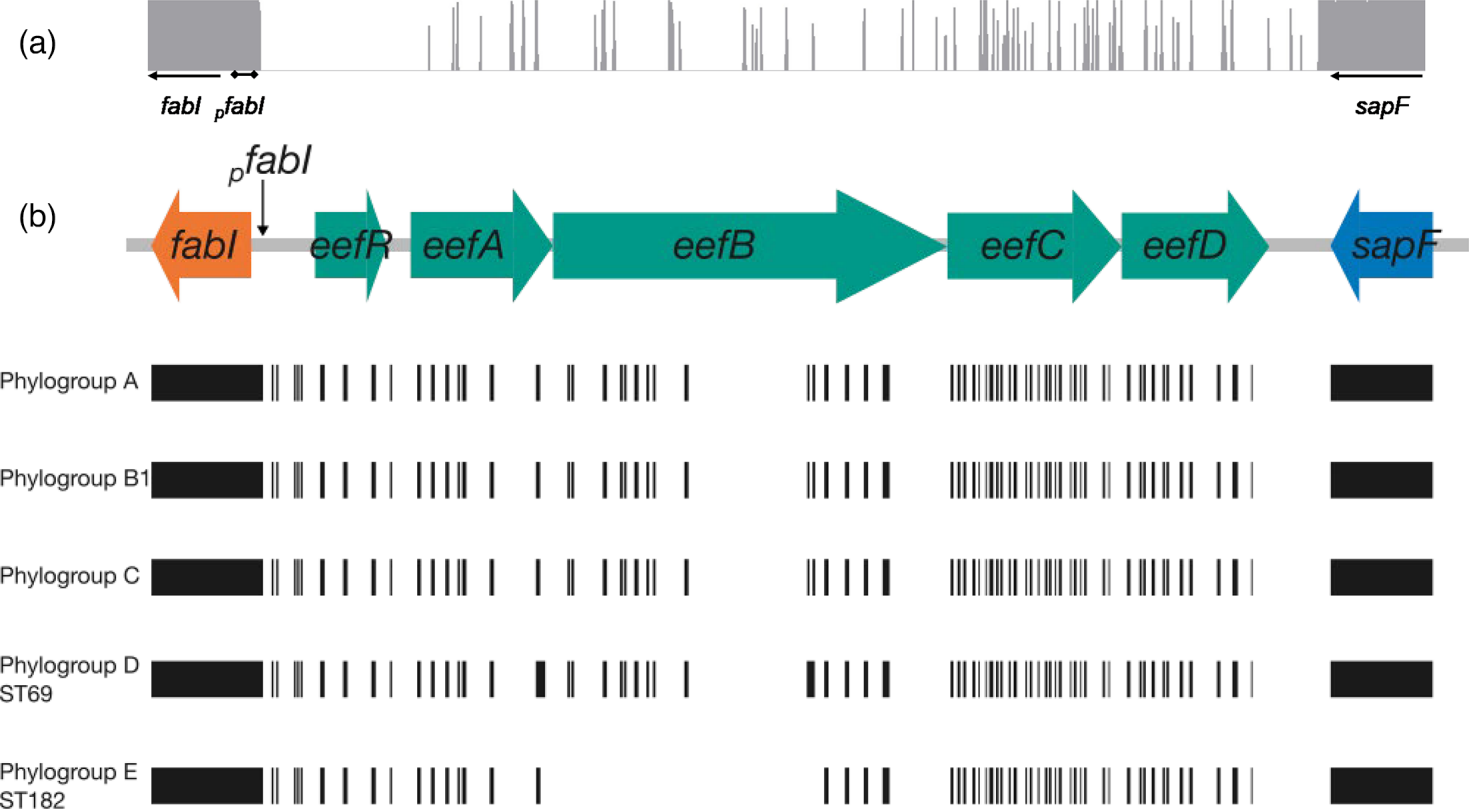
Diagrammatic representation of *eefRABCD* homology in strains that lack the operon. In *E. coli* ST positive for *eefRABCD*, the operon was consistently located between *fabI* and *sapF*. To determine whether *E. coli* assemblies that lacked the operon had conserved regions of the *eef* operon, the equivalent *fabI-sapF* intergenic region of assemblies negative for *eefRABCD* was aligned with *fabI-eefRABCD-sapF* from *E. coli* ATCC 25922. (**a**) Consensus identity of the *fabI-sapF* intergenic region in *E. coli* that lack the *eef* operon. Between all STs, and despite phylogroup, the *fabI-sapF* intergenic regions aligned to the *eefRABCD* operon in a highly conserved manner. (**b**) Cartoon representation of operon fragment conservation in *E. coli* ST that lack the *eef* operon. Closer inspection of the alignment (File S1) found that phylogroups A, B1 and C had almost identical *fabI-sapF* intergenic regions, while ST69 and ST189 had marginally different homology patterns. Taken together, these data suggest that the operon may have been lost in up to three independent events.

A larger alignment of the 97 assemblies from phylogroups A, B1 and C (and ST69 and ST182) used to construct the phylogenetic tree demonstrated that the *ycjD* hypothetical gene is highly conserved across phylogroups A, B1 and C. The alignment of the *sapF-ycjD-fabI* region also demonstrated that ST69 and ST182 possess an intergenic region highly homologous to the STs belonging to phylogroups A, B1 and C, though subtle differences were present (Fig. 3). The ORF annotated as *ycjD* in K-12 is 12 nt longer in both ST69 and ST182 assemblies (Fig. 3). Taken together, these findings suggest that *eef* has been lost from *E. coli* on at least two independent instances.

### Distribution of *eefRABCD* in Gram-negative bacteria

To see if the *eefRABCD* operon was present more widely across *Escherichia* species, the ATCC 25922 *eef* operon was aligned to sequences from the *Escherichia* genus (taxid 561), with *E. coli* (taxid 562) sequences excluded, using the NCBI RefSeq database. Only *E. marmotae* and *E. albertii* were found to possess the *eefRABCD* operon (Fig. S1) while *E. fergusonii*, *Escherichia ruysiae*, *Escherichia vulneris and E. hermannii* did not encode it.

Due to the genetic similarity of *Shigella* and *E. coli* [68,69], the presence of the *eef* operon across *Shigella* spp. was investigated. Gene fragments were detected in a small number of *S. boydii* and *S. flexneri* assemblies but only *S. dysenteriae* was found to consistently possess the operon (Fig. S2, Table S5). However, while genes belonging to the *eefRABCD* operon were detected in 363 of the 486 *S*. *dysenteriae* assemblies included in this study, only a single assembly was positive for *eefR*. Moreover, while *eefA* was present in all assemblies possessing the operon, sequence coverage averaged at 24.6%. Assembly annotation identified the presence of an insertion sequence in the place of *eefR* and *eefA* (Fig. S3) explaining the absence and truncation of *eefR* and *eefA* respectively, across the *S. dysenteriae* assemblies. In comparison, no insertion sequences were detected within the *eef* operon of *E. coli* ATCC 25922, nor in the 10,000 bp up- or downstream of the operon.

As the EefABC efflux pump has been reported in both *K. aerogenes* and *K. pneumoniae* [31,33] with differing operon architecture (Fig. S4), the presence of the operon across related Gammaproteobacterial genera was determined. Only low homology orthologues of EefA and EefB were identified in *Yersinia* and *Serratia*, while no conserved homologues were identified in *Salmonella*, *Acinetobacter*, *Pseudomonas* or *Photorhabdus* (Table S6). In *Enterobacter,* conservation of the operon differed between species (Fig. S5). Some species, such as *E. chengduensis*, encoded *eefRABC*, but not *eefD*; however sequence identity was only 73% (blastn), indicating that the *E. coli* and *Enterobacter eef* operons are not homologous, and instead the association is likely due to historical literature and gene nomenclature.

### Sequence and structural prediction analysis reveals unique features of the EefABC pump

The *eefABC* operon appears to encode a tripartite RND-efflux pump similar to the AcrAB-TolC assembly in *E. coli*. As there is no experimental structural information currently available on any of the components of EefABC, we conducted sequence analysis to identify potential structural templates and subsequently performed homology modelling using the highest scoring templates.

**Fig. 4.**
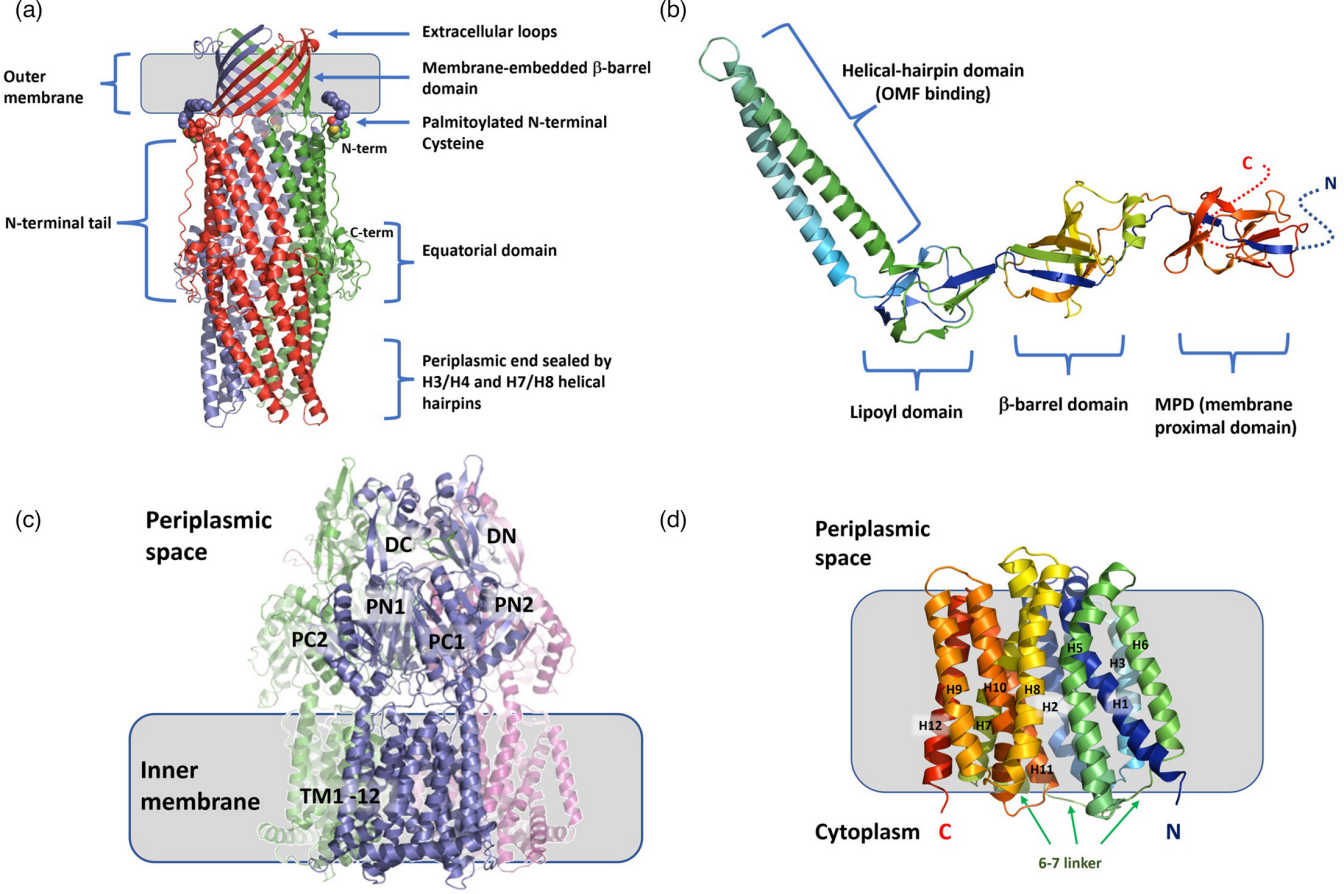
Multi-panel representation of the Eef components. Not to scale. (**a**) A model of the trimer of the EefC trimer, with individual protomers being coloured in red, blue and green, respectively. The palmitoylated N-terminal cysteine residue is shown in space-fill representation. Key structural elements are highlighted. (b) A model of the monomer of EefA, with notations of the individual domains. Low confidence N- and C-terminal regions are represented with dotted lines. (c) A model of the EefB trimer with individual subunits being coloured blue, green and salmon, respectively. The principal subdomains of the periplasmic porter domain of the EefB are annotated (DN, distal N-terminal subdomain; DC, distal C-terminal subdomain; PN, proximal N-terminal subdomain; PC, proximal C-terminal subdomain), alongside the transmembrane helices (TM) 1 to 12. (d) A model of EefD transporter. Individual transmembrane helices are numbered from 1 to 12. The extended linker between the two lobes of the transporter, connecting helices 6 and 7, is also shown.

Comparison with other OMFs of known structure revealed the closest relatives within the wider OMF family to be the *Pseudomonas* proteins OprM and OprJ (Fig. S6), followed by CusC, and hence, OprM/J were used as structural templates for homology modelling (Figs S7 and S8 and supplementary text). Structural alignment of EefC with OprM/J produced an alignment with very few gaps, allowing for direct mapping of the aligned sequences of EefC onto OprM/J. EefC has significantly shorter extracellular loops in comparison to TolC, in particular the L2 loop which occludes TolC opening [70], suggesting a more-open state of the EefC channel (Fig. 4a). Such OMF loops are prominent targets of protective antibodies [71], and the non-protruding loops may serve to avoid antibody restriction and LPS occlusion, thus potentially linking this architecture to the virulence-associated mechanism. Additionally, the N-terminal tail of EefC is 30 residues longer than that seen in TolC, making it more similar to the structure of OprN/J (Figs S8 and S9). A table of similarity/identity matrices for all the sequences used is provided as Table S7.

The comparison of the gating-loop regions in EefC and TolC reveals that they are highly divergent, indicating a locking mechanism markedly different from that observed in TolC, and hence, EefC is unlikely to function with any PAPs that normally pair with TolC and likely only interacts with its cognate PAP EefA. Specifically, several key residues in the helix 7/helix 8 hairpin of TolC, responsible for gating the TolC channel, are different in EefC, e.g. the R367 (TolC), forming part of the so-called ‘primary gate’ that seals the TolC channel by binding to the conserved D153 (TolC; D206 in EefC) and thus anchoring to the helix 4 [72,73] in EefC is substituted by a small non-polar residue G412, making such interaction impossible (Fig. S8).

In addition, the electrostatic properties of the EefC channel are predicted to be dramatically different to TolC and other OMFs, which is likely to impact heavily the ion and drug selectivity of the channel [74]. Firstly, the so-called ‘secondary gate’ of the channel, formed of the prominently conserved double aspartate ring (D371; D373) which forms the basis of cation-selectivity in TolC [75,76], is fully absent in EefC and instead is substituted by small-hydroxylated residues (T416 and T419), a feature which appears to be unique to EefC. Secondly, EefC possesses additional bulky aliphatic residues L415 and L418, which effectively hydrophobically seal the periplasmic end of the channel (Fig. S8).

Due to the availability of more complete full-length experimental templates, EefA homology models were created using both MexA (2V4D.pdb; 53.85% identity [51]) and AcrA (5V5S.pdb; 50.13% identity [52]) as templates (utilizing both I-TASSER and Swiss-model tools) (Figs 4b and S10). While neither of the models delivered the same level of confidence as those for EefC, the alignments with the known PAP structures are readily interpretable, allowing identification of the protein features. A detailed discussion of the EefA structure is given in the supplementary text with the major findings summarized here.

Alignment of EefA and AcrA results in a direct amino acid match with only two gaps in the alignment, one in the unstructured N-terminal tail and another at position Q221 (EefA), which has a four-residue-long deletion relative to AcrA (Figs 4b and S10). This region corresponds to the C-terminal end of *α*-helix 3, which is flanking the *β*-barrel domain in PAPs; however, it is not predicted to affect RND-binding [77]. Despite overall similarity with MexA/AcrA, there are distinctive differences in the organization of the EefA, notably in its *α*-hairpin domain (Figs 4b and S11). While the RLS(D) motif, which is thought to be critical for PAP-OMF interaction [10,78, 79] appears to be preserved in EefA, (R120/L124/S131/D136), there are a number of significant changes in the adjacent residues, notably R123 (K131 in AcrA), V125 (L133) and D134 (E142), which would likely result in steric clashes that would preclude direct compatibility with TolC, and such residues are likely to be playing a discriminatory role engaging with EefC (Fig. S11).

The RND-transporter component, EefB, is a large integral membrane protein, predicted to form a functional trimer similar to other transporters in the family (Fig. 4c). Alignment of the EefB with other RND transporters of known function including AcrB (*E. coli*), MtrD (*N. gonorrhoeae*), MexB (*Pseudomonas aeruginosa*), CusA (*Campylobacter jejuni*) and AdeB (*Acinetobacter baumannii*) revealed that they are highly similar, with EefB being the most similar to AcrB and MexB (57.0% and 56.8% identity respectively), while CusA and MtrD were most divergent (Fig. S12). The MexB structure was used as a template to generate a high-fidelity homology model of EefB, as there were fewer gaps in the alignment (0.5% vs 0.7% for AcrB) and slightly higher residue overlap with EefB (1,038- vs 1,033-residue overlap, respectively).

Overall, the alignment of EefB with MexB (and AcrB) produces very few gaps (the longest is 2 residues long), allowing for unequivocal attribution of secondary structure elements (Fig. S12). As can be seen from the side-by-side comparison of the EefB and AcrB, both present a virtually identical architecture (Fig. S13), with the only notable differences being the shortened loop connecting the TM helices *α*16 and *α*17 in EefB (residues 498–507) and the shorter C-terminal tail. Consistent with this, the critical proton-relay residues found in MexB D407, D408, K939 and T976 [80] are conserved in EefB (D408, D409, K935 and T972, respectively) and the predicted structures of the access and distal binding pockets of EefB suggest a closer relation to the MexB/AcrB than to MexY-type transporters.

The last member of the operon is EefD, a member of the MFS family of efflux pumps that according to the Transporter Classification Database (TCDB) belongs to the 2.A.1.2 group of MFS transporters, related to MDR-function, which also includes Bcr/CflA, EmrD and MdfA [81,82] (Fig. S14). This resulted in high-confidence homology models (I-TASSER C-score=1.82), revealing a classic 6+6 transmembrane helical arrangement closely related to the general topology of MdfA, although with significant differences in the substrate binding cavity, suggesting the substrate range will be notably different between EefD and MdfA (Figs 4d and S15) [60,83, 84]. These discrepancies make predictions of the possible substrates of EefD problematic, although some overlap with MdfA can be expected [85], e.g. lipophilic cations such as ethidium.

**Fig. 5.**
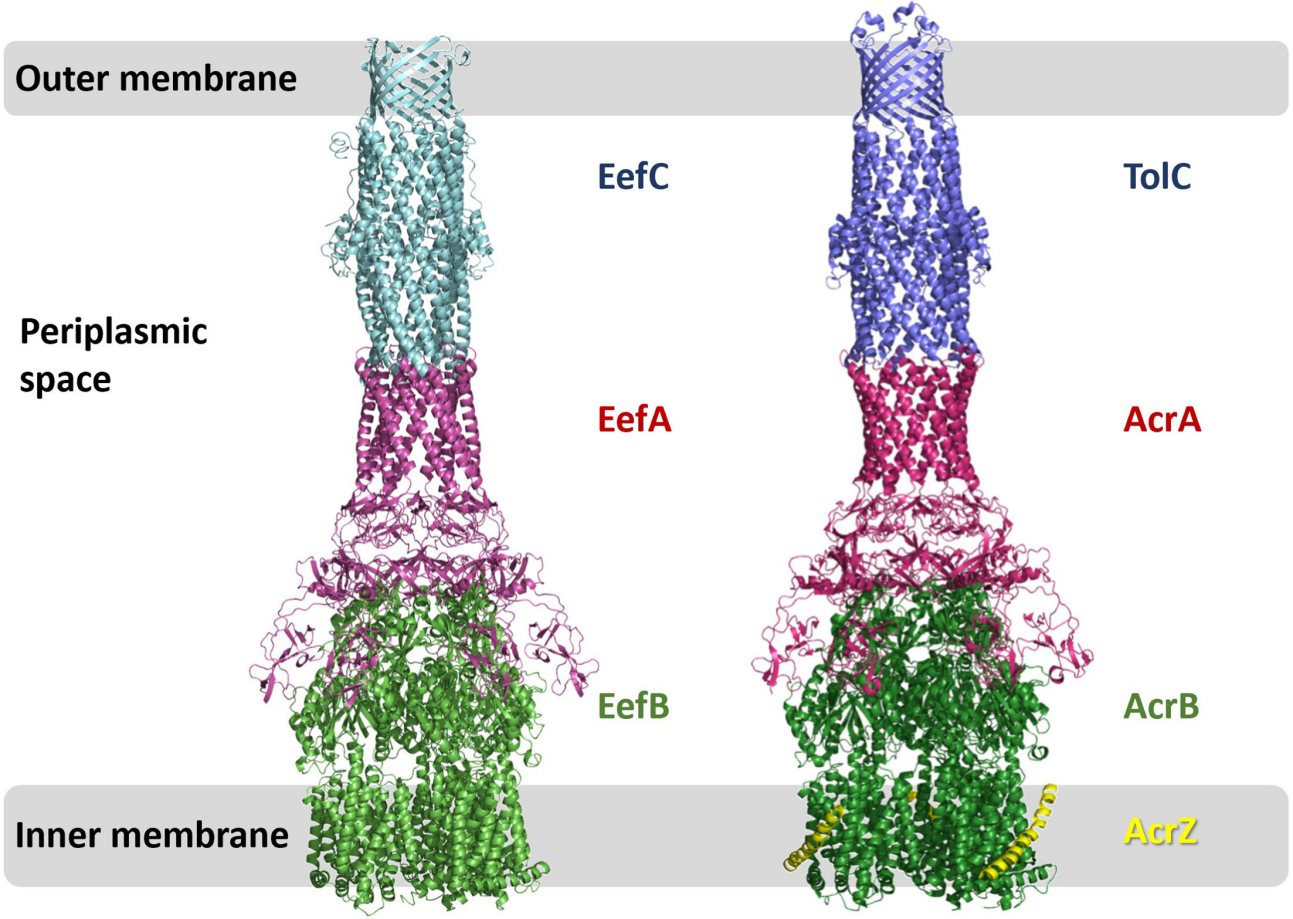
Comparison of the predicted structures of the assembled EefABC and the experimental cryo-EM structure of AcrABZ-TolC (based on 5066.pdb [52]).

We used the homology models described above to dock the components into a complete tripartite pump using the cryo-EM structures of AcrAB-TolC [52] as a guide. EefABC can indeed be assembled using the same architecture with minimal steric clashes, as can be seen in Fig. 5.

### *E. coli* EefABC is not a drug transporter

The high levels of conservation of the *eefRABCD* operon within clinically relevant lineages of *E. coli* suggest it has an important biological function, and its substrate profile may differ from other *E. coli* RND pumps due to its unique structure. Antimicrobials are known substrates of RND efflux pumps such as AcrB, and overexpression of RND pumps can confer MDR in both the laboratory and clinic. Therefore, the effect of *eefABC* and *eefD* expression on *E. coli* susceptibility to a range of antimicrobials and dyes was determined. Due to the previously mentioned homology between *E. coli* ATCC 25922 and *E. coli* SMS-3-5, and the well-characterized antimicrobial susceptibility profile of ATCC 25922, this strain was used for EefABC characterisation experiments.

Deletion of *eefB* in *E. coli* ATCC 25922 did not increase susceptibility to any antimicrobial or dye tested. Subsequent inactivation of *eefA* and *eefC* to give an *eefABC* knockout also had no effect on the susceptibility of *E. coli* ATCC 25922 to antibiotics (Table 2).

**Table 2.** Susceptibility of *E. coli* to antimicrobials following loss and gain of *eefABC* and *eefD*

	Minimum inhibition concn (µg ml^−1^)																		
Strain	AZT	BAC	CAR	CEF	CHL	CIP	CLI	ERY	EB	FA	GEN	MER	MOX	**NA**	NOV	RIF	SPE	TET	TIC
ATCC 25922	0.12	32	16	0.06	4	0.03	128	64	512	512	0.5	0.016	0.03	2	32	>32	8	1	8
ATCC 25922 ΔeefB	0.12	32	16	0.06	4	0.016	128	64	512	512	0.5	0.016	0.03	2	32	>32	8	1	8
ATCC 25922 ΔeefABC	0.25	32	16	0.06	2	0.03	128	64	512	512	0.5	0.016	0.03	2	32	>32	8	1	8
ATCC 25922 ΔeefD	0.12	32	16	0.06	8	0.06	256	64	512	1024	0.5	0.016	0.06	8	64	>32	8	1	8
MG1655+pET21 a	0.12	64	>1,024	0.06	8	0.016	128	32	1024	512	0.12	0.03	0.06	8	128	>32	4	1	>1,024
MG1655+pET21 a eefABC	0.12	64	>1,024	0.06	8	0.016	128	32	1024	1024	0.25	0.03	0.06	8	128	>32	4	1	>1,024
MG1655+pET24 a	0.06	64	8	0.06	8	0.016	128	32	1024	512	0.25	0.03	0.03	8	128	>32	8	1	4
MG1655+pET24 a eefABC	0.06	64	8	0.06	8	0.016	128	32	1024	512	0.25	0.016	0.06	8	128	>32	4	1	4
MG1655+pACYC177	0.06	64	>1,024	0.06	8	0.016	128	32	1024	1024	0.5	0.03	0.06	8	128	>32	4	1	>1,024
MG1655+pACYC177 eefD	0.06	64	8	0.06	8	0.016	128	32	1024	1024	0.5	0.03	0.06	8	128	>32	4	1	4
MG1655 ΔacrB+pET21 a	0.12	4	>1,024	<0.016	1	0.008	2	2	8	8	0.12	0.03	<0.008	2	2	32	4	0.25	>1,024
MG1655 ΔacrB+pET21 a eefABC	0.06	8	>1,024	0.03	1	0.008	2	2	**32**	16	0.12	0.03	<0.008	2	4	32	4	0.25	>1,024
MG1655 ΔacrB+pET24 a	0.12	4	8	<0.016	1	0.008	2	2	8	8	0.25	0.03	<0.008	2	2	32	4	0.25	4
MG1655 ΔacrB+pET24 a eefABC	0.06	8	8	<0.016	2	0.008	2	4	**32**	16	0.25	0.03	<0.008	2	4	32	4	0.25	4
MG1655 ΔacrB+pACYC177	0.12	4	>1,024	<0.016	1	0.008	2	2	8	8	0.5	0.03	<0.008	2	2	32	4	0.25	>1,024
MG1655 ΔacrB+pACYC177 eefD	0.12	4	8	<0.016	1	0.008	2	2	8	8	0.5	0.03	<0.008	2	2	32	4	0.25	4
MG1655 ΔacrB+pET21 a eefABC pACYC177 eefD					2	0.008													

AZT, aztreonam; BAC, benzalkonium chloride; CAR, carbenicillin; CEF, cefotaxime; CHL, chloramphenicol; CIP, ciprofloxacin; CLI, clindamycin; EB, ethidium bromide; ERY, erythromycin; FA, fusidic acid; GEN, gentamicin; MER, meropenem; MOX, moxifloxacin; NA, nalidixic acid; NOV, novobiocin; RIF, rifampicin; SPE, spectinomycin; TET, tetracycline; TIC, ticarcillin.

As loss of EefABC function did not alter the drug susceptibility profile of ATCC 25922, *eefABC* was cloned into the pET21a and pET24a plasmids and expressed in *E. coli* MG1655, which does not naturally encode the system. The expression of *eefABC* in *E. coli* MG1655 did not alter susceptibility to any of the antimicrobials tested however AcrB is the dominant RND pump in *E. coli* and can mask phenotypic changes associated with other efflux systems, so it was deleted. The expression of *eefABC* in the absence of *acrB* still did not reveal any significant changes in susceptibility but did result in a decrease in susceptibility to ethidium bromide and rhodamine 6G, suggesting that these dyes can be transported by the pump (Table S8).

The MFS pump EefD was also characterized; however, both inactivation in *E. coli* ATCC 25922 (loss of function) and expression in *E. coli* MG1655 (gain of function) had no effect on antimicrobial and dye susceptibilities. The expression of *eefD* alone in MG1655 Δ*acrB* also had no effect on minimum inhibition concentrations.

A subset of the RND pumps (including CusABC) is known to pump metal ions and form the subfamily of heavy metal efflux (HME)-pumps [86,89]. Due to the structural similarity of EefC to CusC, the susceptibility to heavy metals was also measured. Yet when either *eefB* or *eefABC* was deleted from ATCC 25922, the susceptibility to cobalt, nickel, zinc and iron was not significantly different to the wild-type ATCC 25922.

### EefABC can export ethidium bromide

Ethidium bromide is a well-defined RND efflux substrate of *E. coli*. We used this substrate to confirm the efflux activity of EefABC and EefD. This was achieved by measuring both intracellular accumulation and active efflux of the dye. As well documented in the literature, deletion of *acrB* increased intracellular accumulation and, as expected, expression of the empty vectors (pET21a and pACYC177) did not reduce cellular accumulation of the dye. The expression of *eefD* alone did not impact the final accumulation of ethidium bromide (Fig. S16). The expression of the tripartite RND system, *eefABC*, reduced ethidium bromide accumulation in the absence of AcrB however not to the level of *E. coli* MG1655. The expression of both *eefABC* and *eefD* plasmids simultaneously in the Δ*acrB* background was found to marginally reduce ethidium bromide accumulation further when compared to Δ*acrB* pET21a::*eefABC*; however, this was not statistically significant. Double expression was only found to have significantly reduced accumulation of ethidium bromide in comparison to *E. coli* MG1655 Δ*acrB*+pACYC177. The importance of *eefD* was further shown by directly measuring efflux as deletion of the MFS pump *eefD* in *E. coli* ATCC 25922 was found to significantly increase the time taken for internal fluorescence levels to drop by 50%, indicating a role in ethidium bromide efflux (Fig. S17).

## Discussion

The number of RND pumps present in different Gram-negative bacterial species varies, and there is increasing evidence that prevalence of RND efflux pumps can also vary between isolates of the same species [27,90,93]. Yet it is still broadly assumed that all *E. coli* isolates possess six RND efflux pumps despite recent work showing that not all six pumps are always functional [29,94]. Here, we further demonstrate that the assumption of six RND pumps is inaccurate; STs belonging to the phylogroups of *E. coli* that are most commonly associated with invasive infection (B2, D, E, F and G) encode a seventh, highly conserved RND pump operon (*eefRABCD*), while the operon was completely absent from phylogroups A, B1 and C, which are generally associated with environmental or commensal lifestyles. The level of conservation suggests that EefRABCD has a biologically important function resulting in a high degree of selection pressure, while the distribution in phylogroups commonly associated with infection could suggest that the system has a role in infection or survival in the host environment.

Across phylogroups A, B1 and C, a 354-nt ORF is found in place of *eefRABCD*. This putative gene *ycjD* runs in the opposite orientation to *eefRABCD* and is highly conserved between STs of these phylogroups. This putative gene is also present in ST69 and ST182, which belong to phylogroup D and E respectively, though in these two STs a 12-nt insertion is present at the 3′ end of the gene. Studies from another group support the hypothesis that transcriptional activity is happening at this ORF as public data from their transcriptional start site, term-seq and ribosomal profiling studies show antisense transcriptional and translational activity at the 5′ end of *ycjD* gene [95,97]. It is worth noting that while *ycjD* is indeed coding a domain of unknown function, the UniProtKB AlphaFold structural prediction for it strongly suggests that it is linked to DNA modification/restriction, as it belongs to the endonuclease/DNA methylase fold.

The EefABC efflux pump was first described in the opportunistic human pathogen *K. aerogenes* and subsequently in *K. pneumoniae*, though operon structure differs between the two *Klebsiella* species [31,33]. In *K. aerogenes*, overexpression of *eefABC* decreased susceptibility to erythromycin and ticarcillin [32], while overexpression of *K. pneumoniae eefA* and *eefB* in an *E. coli* (*ΔacrAB* and *ΔydhE*) background decreased susceptibility to oxacillin, erythromycin, novobiocin, acriflavine, ethidium bromide and cholate [98]. However, in this study, neither gain nor loss of *eefABC* function from *E. coli* altered susceptibility to any antibiotics tested, though overexpression did decrease susceptibility to ethidium bromide. Moreover, the expression of *eefABC* (in the absence of AcrB) decreased intracellular accumulation of ethidium bromide, demonstrating that the pump is functional. No effect was seen upon inactivation of the pump, but this is likely because AcrB was still present which would mask any effect. However, deletion of the inner membrane component *eefD* alone caused significantly slower EtBr efflux even in the presence of AcrB, suggesting it has a role in transport of substrates across the inner membrane. The apparent difference in substrate profile between *Klebsiella* and *E. coli* could be due to differences in nucleotide sequence or operon structure subsequently altering the regulation of the operon or function of the orthologous protein. In *K. aerogenes*, the operon has been demonstrated to be H-NS silenced in laboratory conditions [31,32], though due to the genomic location of the *eef* operon in *E. coli* and the resulting proximity of the *eefR* promoter to that of *fabI* which encodes an essential enoyl-[acyl-carrier-protein] reductase, it is unlikely that the operon is silenced *in vivo* in *E. coli*.

When looking across the *Escherichia* genus, *eefRABCD* was only present in *E. marmotae* and *E. albertii*, though the operon was identified in *S. dysenteriae,* which has high genetic similarity to *E. coli*. Here, *eefR* was generally absent due to the presence of an insertion sequence in place of *eefR* and *eefA*. A further insertion sequence was identified downstream of *eefD*. It has been shown previously that *Shigella* species have higher numbers of insertion sequences when compared to other Gammaproteobacteria such as *E. coli* [99]. High numbers of insertion sequences are linked to recent host specification as the integration of these elements into the genomes is associated with early stage genome degradation [100,102]. The presence of an insertion sequence in place of *eefR* and *eefA* and downstream of *eefD* may therefore suggest the operon is in the process of being lost or degraded in *S. dysenteriae*. Many Gram-negative bacteria including *Salmonella* sp. (taxid 590) did not encode an orthologue of the Eef system, but orthologues of EefA and EefB were identified in *Serratia* and *Yersinia* species, suggesting that related systems could be dispersed through the Gammaproteobacteria.

Overall, our analysis of EefABC revealed unexpected similarities to the tripartite pumps OprM-MexAB and OprJ-MexCD in *Pseudomonas* [103], rather than to any endogenous *E. coli* paralogues, which may suggest that these pumps have been acquired via a lateral gene transfer event, similar to some other plasmid-encoded efflux/secretion systems, e.g. the EAEC virulence plasmid pAA2 [104].

Given the close similarities of the EefABC system to both MexAB-OprM and MexCD-OprJ and based on previous profiling of these *Pseudomonas* pumps [103], we initially hypothesized that these pumps could have overlapping substrate ranges. However, despite the overall similarity, our tests did not reveal any direct effect of EefABC on transport of antimicrobials, suggesting alternative function, though it is possible that transport may occur only under specific conditions that have not been tested here [105] and further work will be needed to determine this.

As *E. coli* also possesses HME-RND systems such as CusABC, we also investigated the possibility that EefABC may be involved in metal ion tolerance, but we were unable to identify a metal ion substrate for the efflux pump (Table S8). As mentioned above, the EefC lacks the double aspartate ‘gates’ which have been implicated in the coordination of metal ions in the case of TolC [73,106] (Fig. S8). This alone, however, could not be used to rule out ion-efflux function, as it has previously been noted [107] that CusC does not [108] by itself show any specific features associated with monovalent ion selectivity, with that function rather being associated with the RND transporter, in that case CusA. Indeed, while CusA presents a clear relay of methionine clusters to bind and export Cu(I) and Ag(I) ions [109], there is no identifiable pattern of such residues in the case of EefB. On the other hand, divalent-ion specific pumps such as ZneA have more restricted binding sites formed from negatively charged residues [110], but again, the residues participating in ion coordination are not conserved in EefB.

Aside from conferring resistance to antimicrobials, RND efflux pumps have myriad other physiological roles involving export of both exogenous and endogenous substrates [111]. For example, RND efflux pumps in various Gram-negative bacteria have been linked to virulence [17,112]. While we have so far been unable to assign clear function to EefRABCD, given the striking distribution and conservation of *eefRABCD* only in phylogroups of *E. coli* associated with disease, it is possible that this pump has roles associated with virulence or survival in the host environment, and work is continuing to assign biological function.

## Supplementary material

10.1099/mgen.0.001593 Supplementary Material 1.

## Abbreviations

Cryo-EM: cryogenic-electron microscopy
HME: heavy metal efflux
MDR: multidrug resistance
MFS: major facilitator superfamily
MSA: multiple sequence alignment
OMF: outer membrane factor
PAP: periplasmic adaptor protein
RND: resistance-nodulation-division
ST: sequence type
