# Supplementary material for: The Resistance-Nodulation-Division efflux pump EefABC is highly conserved within lineages of E. coli commonly associated with infection
Source: Microb Genom. 2026 Jan 12;12(1):001593. doi: 10.1099/mgen.0.001593 (PMC12817139; doi:10.1099/mgen.0.001593)
Supplement: Supplementary Material 1. [file mgen-12-01593-s001.pdf]

Table S1. Strains used in this study.

Species	Genotype	Marker	Reference
E. coli	CGSC - DB Strain K-12 MG1655		(Blattner *et al.*, 1997)
E. coli	K-12 MG1655 Δ*acrB*		(Wang-Kan *et al.*, 2017)
E. coli	ATCC 25922		PHE
E. coli	ATCC 25922 *eefB*::*aph*	KanR	This study
E. coli	ATCC 25922 Δ*eefB*		This study
E. coli	ATCC 25922 *eefABC*::*aph*	KanR	This study
E. coli	ATCC 25922 Δ*eefABC*		This study
E. coli	ATCC 25922 *eefD*::*aph*	KanR	This study
E. coli	ATCC 25922 Δ*eefD*		This study
E. coli	K-12 MG1655 pACYC177	AmpR KanR	This study
E. coli	K-12 MG1655 + pACYC177 *eefD*	KanR	This study
E. coli	K-12 MG1655 *ΔacrB* pACYC177	AmpR KanR	This study
E. coli	K-12 MG1655 Δ*acrB* pACYC177 *eefD*	KanR	This study
E. coli	K-12 MG1655 + pET21a	AmpR	This study
E. coli	K-12 MG1655 + pET24a	KanR	This study
E. coli	K-12 MG1655 Δ*acrB* pET24a	KanR	This study
E. coli	K-12 MG1655 + pET21a::*eefABC*	AmpR	This study
E. coli	K-12 MG1655 + pET24a::*eefAB*C	KanR	This study
E. coli	K-12 MG1655 Δ*acrB* + pET21a::*eefABC*	AmpR	This study
E. coli	K-12 MG1655 Δ*acrB* + pET24a::*eefABC*	KanR	This study
E. coli	K-12 MG1655 Δ*acrB* + pET21a::*eefABC* + pACYC177 *eefD*	AmpR KanR	This study

Table S2. Primers used in this study.

Name	Description	Primer sequence 5'-3'
eefABC cloning forward	Amplifies *eefABC* from the *E. coli* ATCC 25922 chromosome incorporating the *NdeI* restriction site for pET21a/pET24a ligation	TTGACCATATGAAGTATATAGCAACATCTGTAGTG
eefABC cloning reverse	Amplifies *eefABC* from the *E. coli* ATCC 25922 chromosome incorporating the *XhoI* restriction site for pET21a/pET24a ligation	GTGGTGCTCGAGTTACTTGAGTGATGACATACCGCCG
eefD cloning forward	Amplifies *eefD* from the *E. coli* ATCC 25922 chromosome incorporating the *ApaLI* restriction site for pACYC177 ligation	CCCCCCGTGCACATGGCTAGAGTCTCTCTTTCATGGGCATTGATTCTTGG
eefD cloning reverse	Amplifies *eefD* from the *E. coli* ATCC 25922 chromosome incorporating the *PstI* restriction site for pACYC177 ligation	CCCCCCCTGCAGTTACTTTTGATTGTCGATTCTGGTGAGAGCAAC
eefB deletion forward	Amplifies the *aph* cassette from pKD4 incorporating *eefB*	AGGCTGTTGAAGTTCAGGCTAATGGAGGCAACGCCTGATGTTTgtgtaggctggagctgcttc

eefB deletion reverse	overhangs for interruption of *eefB* on the *E. coli* ATCC 25922 chromosome	ACAACACCAGAAAAATAAGAGAACGACGCAACATAACTTAgggaattagccatggtccat
eefAC deletion forward	Following the inactivation of *eefB* and removal of the *aph* cassette, these primers amplify the *aph* cassette with *eefA* and *eefC* overhangs from the inactivation of the remaining region	TTCAATGAGGAGGGTATTTCTCACCCTGGAGAAAATAATGgtgtaggctggagctgcttc
eefAC deletion reverse		AATGCCCATGAAAGAGAGACTCTAGCCATATTATTCCTTAgggaattagccatggtccat
eefD deletion forward	Amplifies *aph* from the pKD4 plasmid, enabling the inactivation of *eefD,* independent of *eefABC*	CTGGGCGGCGGTATGTCATCACTCAAGTAAGGAATAATgtgtaggctggagctgcttc
eefD deletion reverse		GCACCTCATCCTGACAGGCCCCCCTCAGGATGAGGTGTgggaattagccatggtccat

Table S3. The number of *E. coli* assemblies downloaded, the number of assemblies removed due to being duplicate assemblies or due to *eef* being split over multiple contigs, and the final number of assemblies used in this study.

Phylogroup	ST	Downloaded assemblies	Duplicates removed	Split operons removed	Final assemblies
A	ST10	1758	0	1	1757
	ST119	110	0	0	110
	ST167	115	0	0	115
	ST540	136	14	0	122
	ST617	181	0	0	181
B1	ST17	1884	0	0	1884
	ST21	2411	0	0	2411
	ST53	5	0	0	5
	ST84	2	0	0	2
	ST156	210	10	0	200
	ST328	54	0	0	54
	ST453	142	6	0	136
	ST655	856	238	1	617
	ST678	116	9	0	107
B2	ST12	479	41	3	435
	ST14	46	0	0	46
	ST73	916	0	7	909
	ST95	751	0	0	751
	ST127	231	0	1	230
	ST131	3186	0	30	3156
	ST144	85	0	0	85
C	ST23	384	19	0	365
	ST88	454	33	0	421
	ST90	124	8	0	116
	ST410	596	23	2	571
D	ST38	815	29	10	776
	ST69	696	0	0	696
	ST405	525	35	9	481
	ST963	55	2	0	53
E	ST11	1490	132	30	1328
	ST182	76	1	3	72
	ST350	34	4	1	29
	ST1266	15	0	2	13
F	ST59	175	18	1	156
	ST62	217	122	2	93
	ST354	266	22	7	237
	ST648	379	0	1	378
G	ST738	38	0	0	38

Table S4. Presence and subsequent gene coverage of *eefRABCD* across the *E. coli* assemblies with the *eefRABCD* operon of *E. coli* ATCC used as a reference.

Phylogroup	ST group	Assemblies (*n*)	Mean sequence coverage to ATCC 25922 *eef* operon (%)				
			eefR	***eefA***	***eefB***	***eefC***	***eefD***
A	10	1757	0.06	0.06	0.06	0.06	0.00
	119	110	0.00	0.00	0.00	0.00	0.00
	167	115	0.00	0.00	0.00	0.00	0.00
	540	122	0.00	0.00	0.00	0.00	0.00
	617	181	0.55	0.55	0.55	0.55	0.55
B1	17	1884	0.00	0.00	0.00	0.00	0.00
	21	2411	0.00	0.00	0.00	0.00	0.00
	53	5	0.00	0.00	0.00	0.00	0.00
	84	2	0.00	0.00	0.00	0.00	0.00
	156	200	0.50	0.00	0.00	0.00	0.00
	328	54	0.00	0.00	0.00	0.00	0.00
	453	136	0.00	0.00	0.00	0.00	0.00
	655	617	0.00	0.00	0.00	0.00	0.00
	678	107	0.00	0.00	0.00	0.00	0.00
B2	12	435	100.00	99.84	99.77	99.77	100.00
	14	46	100.00	100.00	100.00	100.00	100.00
	73	909	100.00	100.00	100.00	100.00	99.97
	95	751	99.99	100.00	100.00	100.00	100.00
	127	230	99.98	100.00	100.00	100.00	100.00
	131	3156	99.87	99.87	99.91	99.91	99.95
	144	85	99.99	100.00	100.00	100.00	100.00
C	23	365	0.27	0.27	0.08	0.00	0.00
	88	421	0.00	0.00	0.02	0.00	0.00
	90	116	0.00	0.00	0.00	0.00	0.00
	410	571	0.00	0.00	0.00	0.00	0.00
D	38	776	99.69	99.74	99.74	99.74	99.74
	69	696	0.00	0.00	0.00	0.00	0.00
	405	481	99.97	100.00	100.00	100.00	100.00
	963	53	99.99	100.00	100.00	100.00	100.00
E	11	1328	99.99	100.00	99.90	100.00	100.00
	182	72	0.00	0.00	0.00	0.00	0.00
	350	29	99.68	100.00	100.00	100.00	100.00
	1266	13	100.00	100.00	100.00	100.00	100.00
F	59	156	99.32	99.36	99.36	99.21	99.36
	62	93	98.92	98.91	98.92	98.92	98.92
	354	237	98.50	99.07	99.16	99.16	99.35
	648	378	100.00	100.00	100.00	100.00	100.00
G	738	38	100.00	100.00	100.00	100.00	100.00

Table S5. Presence and subsequent gene coverage and nucleotide identity of *eefRABCD* across four *Shigella* species with reference to the *E. coli* ATCC 25922 (CP009072.1) *eefRABCD* operon

Species	*n*	Assemblies positive for an *eef* gene	*eefR*			*eefA*			*eefB*			*eefC*			*eefD*		
			n	% ID	% coverage	*n*	% ID	% coverage	*n*	% ID	% coverage	*n*	% ID	% coverage	*n*	% ID	% coverage
Shigella boydii	469	15	14	99.22	100.00	14	97.95	100.00	15	96.63	62.36	9	97.32	100.00	14	97.89	83.10
Shigella dysenteriae	486	363	1	98.94	99.82	363	97.62	26.40	363	98.29	100.00	363	97.38	100.00	363	97.25	98.97
Shigella flexneri	352	1	1	99.12	100.00	1	97.95	100.00	1	98.17	100.00	1	97.53	100.00	1	98.37	100.00
Shigella sonnei	360	0	0	0	0	0	0	0	0	0	0	0	0	0	0	0	0

Percentages shown are means.

Table S6. Conservation of *eefB* homologs across five closely related bacterial genera.

		Genome									
		1		2		3		4		5	
Genus	Taxid	Coverage (%)	Identity (%)	Coverage (%)	Identity (%)	Coverage (%)	Identity (%)	Coverage (%)	Identity (%)	Coverage (%)	Identity (%)
Yersinia	629	98	68.47	98	68.47	98	68.52	98	68.52	98	68.49
Serratia	613	99	69.76	92	72.89	85	72.89	92	72.82	86	72.82
Pseudomonas	286	13	98.08	31	74.03	29	75.84	30	75.63	38	75.63
Photorhabdus	29487	25	72.43	25	72.04	25	72.04	25	72.04	36	71.84
Salmonella	590	44	74.89	47	74.89	53	74.89	32	76.01	36	75
Acinetobacter	469	98	68.49	22	70.68	10	77.36	10	76.76	32	70.88

Table S7. **Similarity and identity scores for the templates discussed in text calculated using EMBOSS NEEDLE (https://www.ebi.ac.uk/jdispatcher/psa/emboss_needle), based on the respective full-length UNIPROT sequences.**

	Identity	Similarity	Gaps	Score
	**EefC**			
**OprJ_PSEAE_Q51397**	**177/489 (36.2%)**	**263/489 (53.8%)**	**49/489 (10.0%)**	**733.5**
**OprM_PSEAE_Q51487**	203/494 (41.1%)	290/494 (58.7%)	53/494 (10.7%)	893.5
**OprN_PSEAE_Q9I0Y7**	136/495 (27.5%)	221/495 (44.6%)	68/495 (13.7%)	429.5
**CusC_ECOLI_K12_P77211**	**163/465 (35.1%)**	**262/465 (56.3%)**	**23/465 (4.9%)**	**694.5**
**TolC_K12_P02930**	106/573 (18.5%)	187/573 (32.6%)	203/573 (35.4%)	224.0
	**EefA**			
**AcrA_ECOLI_P0AE06**	188/381 (49.3%)	247/381 (64.8%)	13/381 (3.4%)	875.0
**MexA_PSEAE_P52477**	197/401 (49.1%)	254/401 (63.3%)	46/401 (11.5%)	864.0
	**EefB**			
**AcrB_ECOLI_P31224**	591/1053 (56.1%)	774/1053 (73.5%)	22/1053 (2.1%)	3026.5
**MexB_PSEAE_P52002**	590/1047 (56.4%)	764/1047 (73.0%)	13/1047 (1.2%)	3015.0
**CusA_ECOLI_P38054**	265/1127 (23.5%)	484/1127 (42.9%)	172/1127 (15.3%)	744.5
**AdeB_Q93E19_ACIBA**	482/1047 (46.0%)	695/1047 (66.4%)	24/1047 (2.3%)	2367.5
**MexY_Q9ZNG8_PSEAI**	460/1063 (43.3%)	666/1063 (62.7%)	45/1063 (4.2%)	2219.0
	**EefD**			
**EmrD_ECOLI_P31442**	110/413 (26.6%)	200/413 (48.4%)	45/413 (10.9%)	377.0
**MdfA_ECOLI_P0AEY8**	102/423 (24.1%)	178/423 (42.1%)	49/423 (11.6%)	279.0
**EmrD_2GFP.pdb**	106/409 (25.9%)	191/409 (46.7%)	56/409 (13.7%)	370.5
**MdfA_4ZP0.pdb**	100/417 (24.0%)	173/417 (41.5%)	55/417 (13.2%)	284.0

Parameters used: Matrix EBLOSUM62; Gap_penalty: 10.0; Extend_penalty: 0.5

Table S8. Susceptibility of *E. coli* to metals, dyes and bile salts

	**MIC (μg/mL)**										
	**Metals**			**Dyes**				**Bile Salts**			**ABX**
**Strain**	**CoCl2**	**CuSO4**	**NiCl**	**CV**	**EB**	**MB**	**R6G**	**SC**	**SDC**	**CDC**	**POL**
ATCC 25922	512	256	512	32	512	>1024	1024	>32,096	32,096	32,096	0.5
ATCC 25922 Δ*eefB*				32	512	>1024	512				
ATCC 25922 Δ*eefABC*	512	256	512	32	512	>1024	512	>32,096	32,096	32,096	0.5
ATCC 25922 Δ*eefD*	512			32	512	>1024	1024				0.5
MG1655 + pET21a				16	1024	>1024	512				
MG1655 + pET21a *eefABC*				32	1024	>1024	512				
MG1655 + pET24a				16	1024	>1024	512				
MG1655 + pET24a *eefABC*				16	1024	>1024	512				
MG1655 + pACYC177				32	1024	>1024	512				
MG1655 + pACYC177 *eefD*				32	1024	>1024	512				
MG1655 Δ*acrB* + pET21a	512	256	512	4	8	8	8	>32,096	16,048	8,024	1
MG1655 Δ*acrB* + pET21a *eefABC*	512	256	512	4	**32**	16	**32**	>32,096	32,096	4,012	0.5
MG1655 Δ*acrB* + pET24a				4	8	8	8				
MG1655 Δ*acrB* + pET24a *eefABC*				4	**32**	16	**32**				
MG1655 Δ*acrB* + pACYC177	512	526	512	4	8	8	8	>32,096	16,048	4,012	0.5

MG1655 Δ*acrB* + pACYC177 *eefD*	512	256	512	4	8	8	8	>32,096	16,048	4,012	0.5
MG1655 Δ*acrB* + pET21a eefABC pACYC177 *eefD*	512	526	512					>32,096	16,048	4,012	0.5

CoCl2 – cobalt chloride CuSO4 – copper sulphate NiCl – nickel chloride CV – crystal violet EB – ethidium bromide MB – methylene blue R6G – rhodamine 6G SC – sodium cholate SDC – sodium deoxycholate CDC – chenodeoxycholate POL – polymyxin B

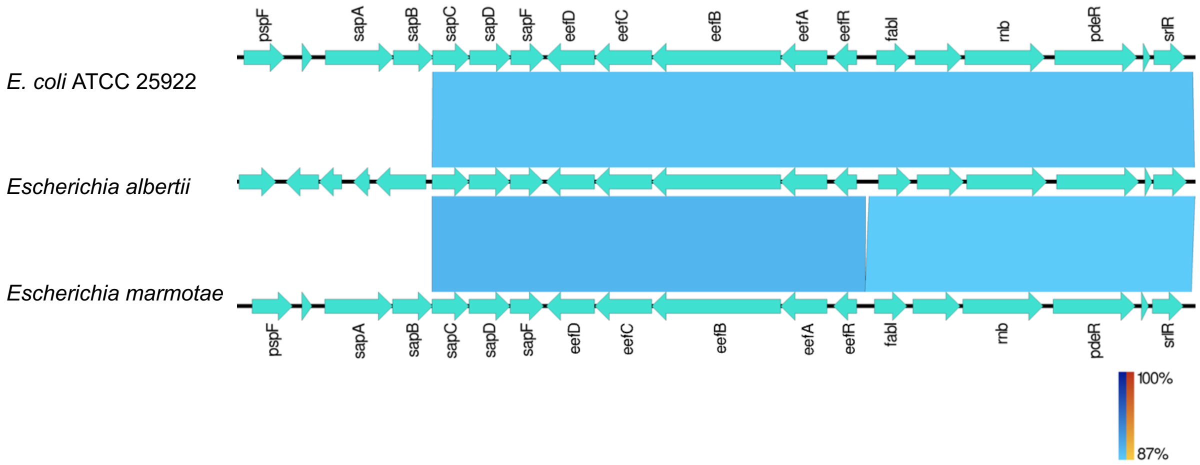

Figure S1. Alignment of the *eef* operon of multiple *Escherichia* species.
As *eefRABC* was found to be highly prevalent across *E. coli,* the wider *Escherichia* genus was searched. Only *E. albertii* and *E. marmotae* were found to possess any of the *eef* genes though conservation was significantly lower than when comparing *E. coli* assemblies. Here, *E. albertii* (CP070290.2) and *E. marmotae* (CP056165.1) are aligned to the *eefRABCD* region of *E. coli* ATCC 25922 (CP009072.1) using blastn and visualised using EasyFig. *E. albertii* and *E. marmotae* were annotated using Prokka.

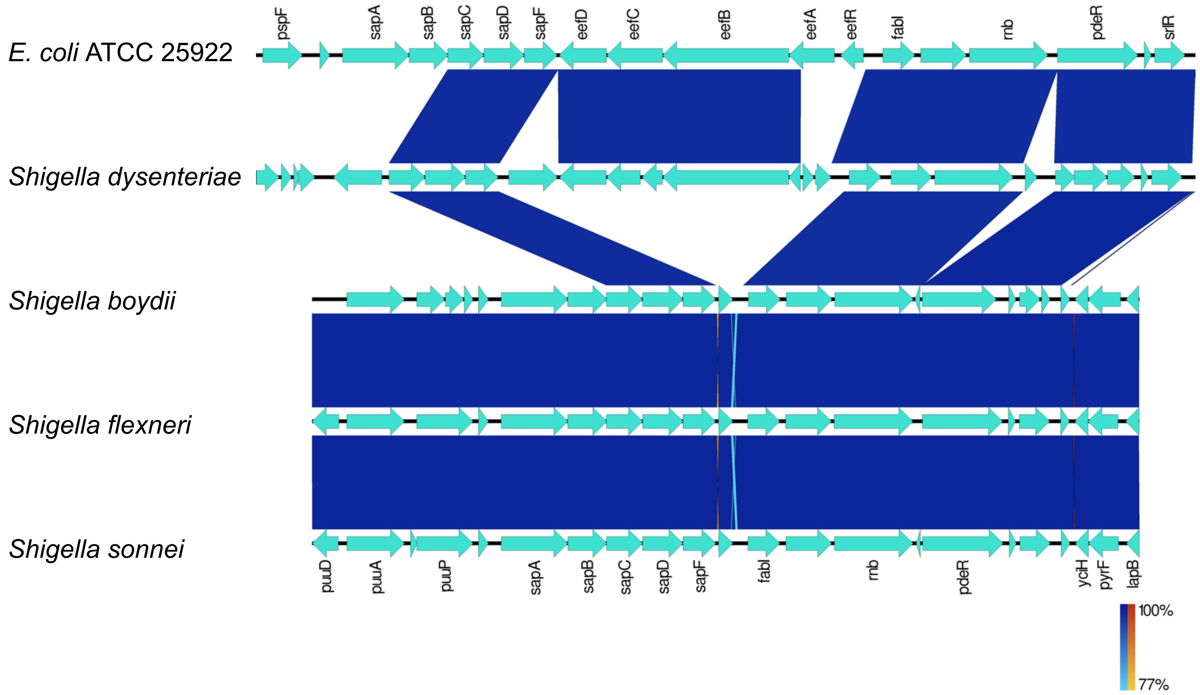

Figure S2. Alignment of *E. coli* ATCC 25922 *eef* operon and corresponding genome region in multiple *Shigella* species.

Shigella species have high genetic similarity to *E. coli,* therefore we looked for the presence of *eefRABCD* across assemblies of *S. boydii* (*n* = 469)*, S. dysenteriae* (*n* = 486)*, S. flexneri* (*n* = 352) and *S. sonnei* (*n* = 360). The operon was completely absent from *S. sonnei.* Only one of the *S. flexneri* assemblies possessed at least one *eef* gene, whilst 15 *S. boydii* assemblies had a gene homologous to the *eefRABCD* operon. However, 363 of the 486 *S. dysenteriae* assemblies were positive for genes homologous to at least of the *eefRABCD* genes however, *eefR* was absent from all assemblies except one. Alignment of four NCBI *Shigella* nucleotide sequences with EasyFig demonstrates the high nucleotide homology between *E. coli* ATCC 25922 (CP009072.1) and *S. dysenteriae* (CP026774.1). *S. boydii* (CP026836.1), *S. flexneri* (AE005674.2) and *S. sonnei* (CP055292.1) all lack the operon but are highly homologous to each other.

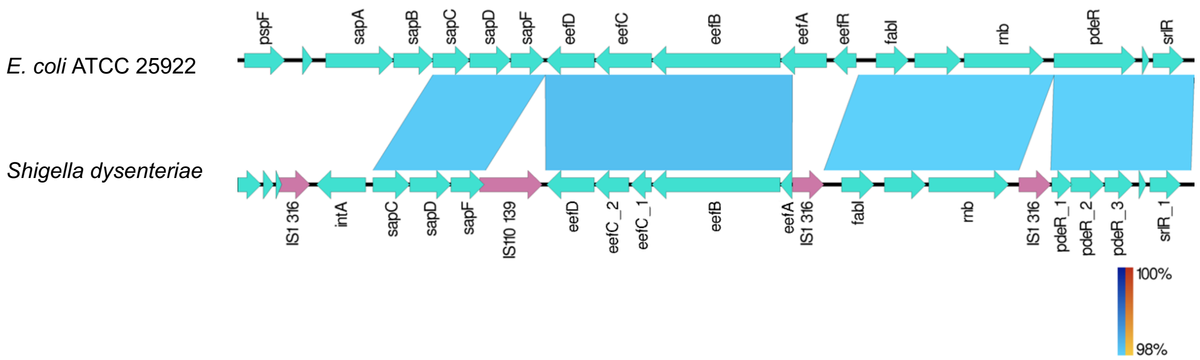

Figure S3. *S. dysenteriae* possesses insertion sequences within the *eef* operon. The presence of *eefRABCD* across *S. boydii, S. flexneri, S. dysenteriae* and *S. sonnei* was identified using ABRicate and a custom blast database generated from the *eefRABC* gene sequences of *E. coli* ATCC 25922 (CP009072.1). This data suggested *eefBCD* were present across *S. dysenteriae* but *eefR* and *eefA* were either completely absent or partially absent. Annotation of *S. dysenteriae* (CP026774.1) with ISEScan identified an IS3 insertion sequence in place of *eefR* and *eefA* and a further IS110 element downstream of *eefD. S. dysenteriae* was annotated using Prokka.

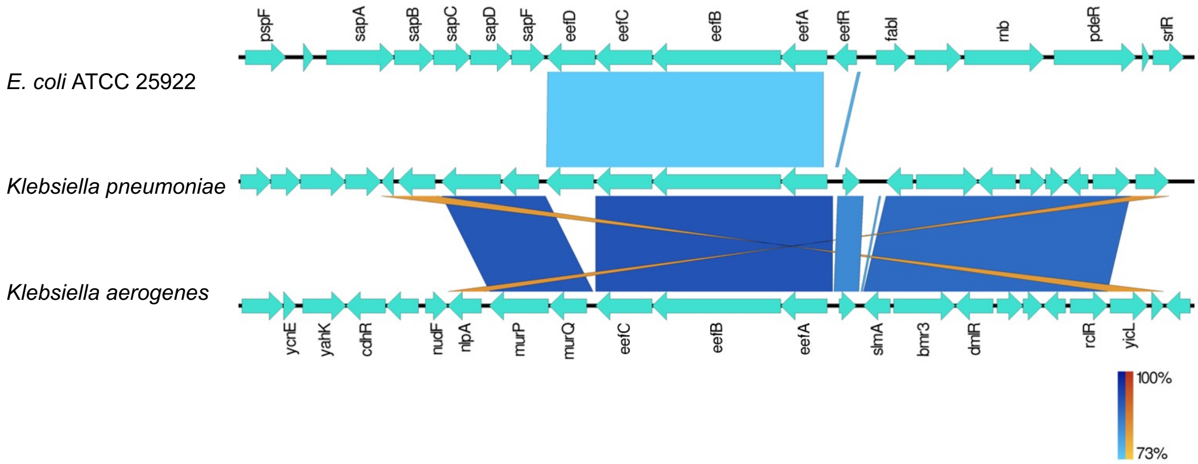

Figure S4. Alignment of the *E. coli* and *Klebsiella* species *eef* operons

The presence of *eef* has been reported in both *K. aerogenes* and *K. pnuemoniae* however in *K. aerogenes* the operon was reported to be H-NS silenced (Masi et al., 2005). Alignment of *K. pneumoniae* (NC_016845.1) and *K. aerogenes* (NZ_CP041925.1) against *E. coli* ATCC 25922 (CP009072.1) demonstrates not only differing operon architecture between the *Klebsiella* species but also a different genome location and reduced nucleotide homology to the *E. coli eef* operon. *K. aerogenes* was annotated using Prokka.

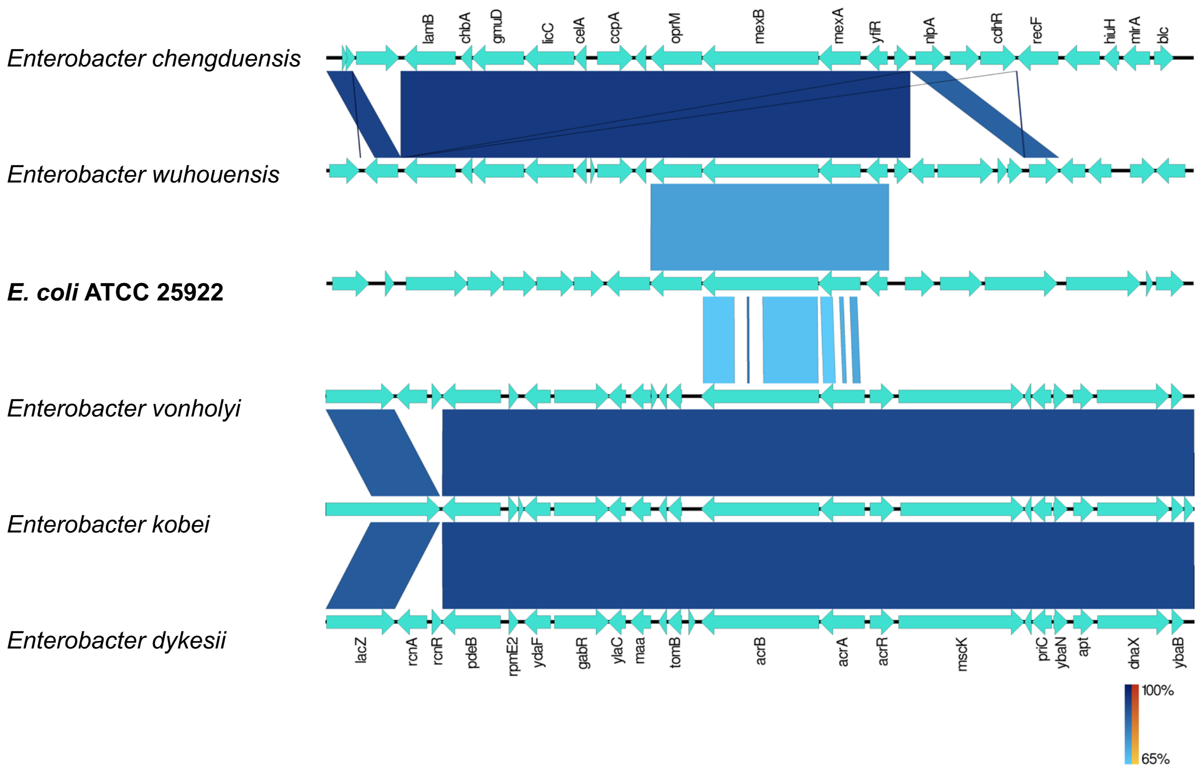

Figure S5. Alignment of *Enterobacter* species against *E. coli* ATCC 25922. Though *E. aerogenes* has since been reclassified as a *K. aerogenes* we looked at the *eef* presence in five *Enterobacter* species. Nucleotide blast was used to identify the position of genes with homology in *E. vonholyi* (VTUC01000001.1), *E. dykesii* (VTTY01000003.1), *E. wuhouensis* (SJOO01000006.1), *E. kobei* (KI973153.1) and *E. chengduensis* (CP043318.1) and these genome regions were then aligned and visualised using EasyFig. A four gene operon was identified in *E. chengduensis* but nucleotide identity was low when compared to *E. coli. Enterobacter* sequences were annotated using Prokka.

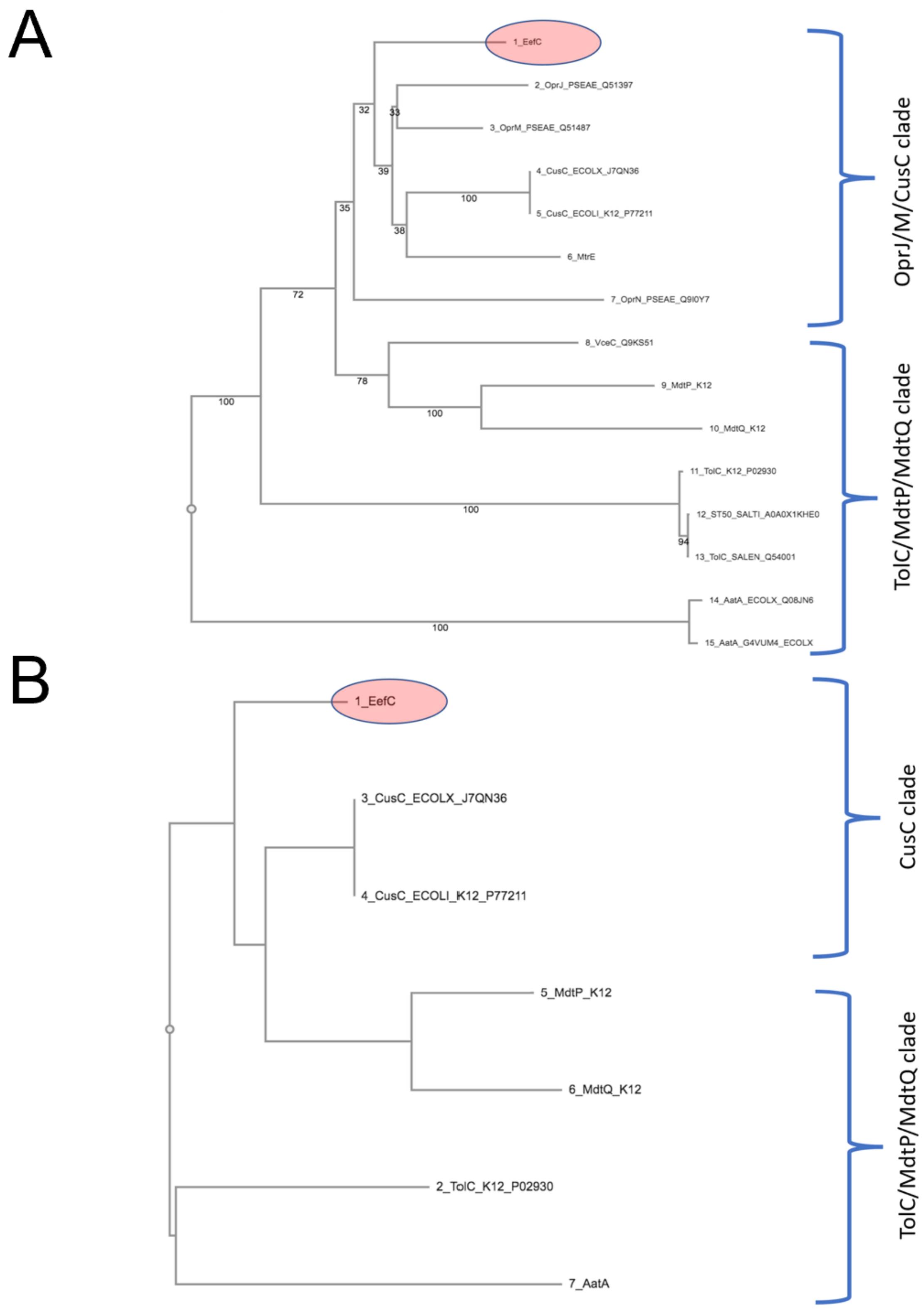

Figure S6. Dendrograms, showing the phylogenetic connections of EefC to the wider OMF family (A) and its relations with the OMFs of *E. coli* (B).
Note the clear connection to the OprM/OprJ clade and the CusC branch, justifying their choice as templates for homology modelling.

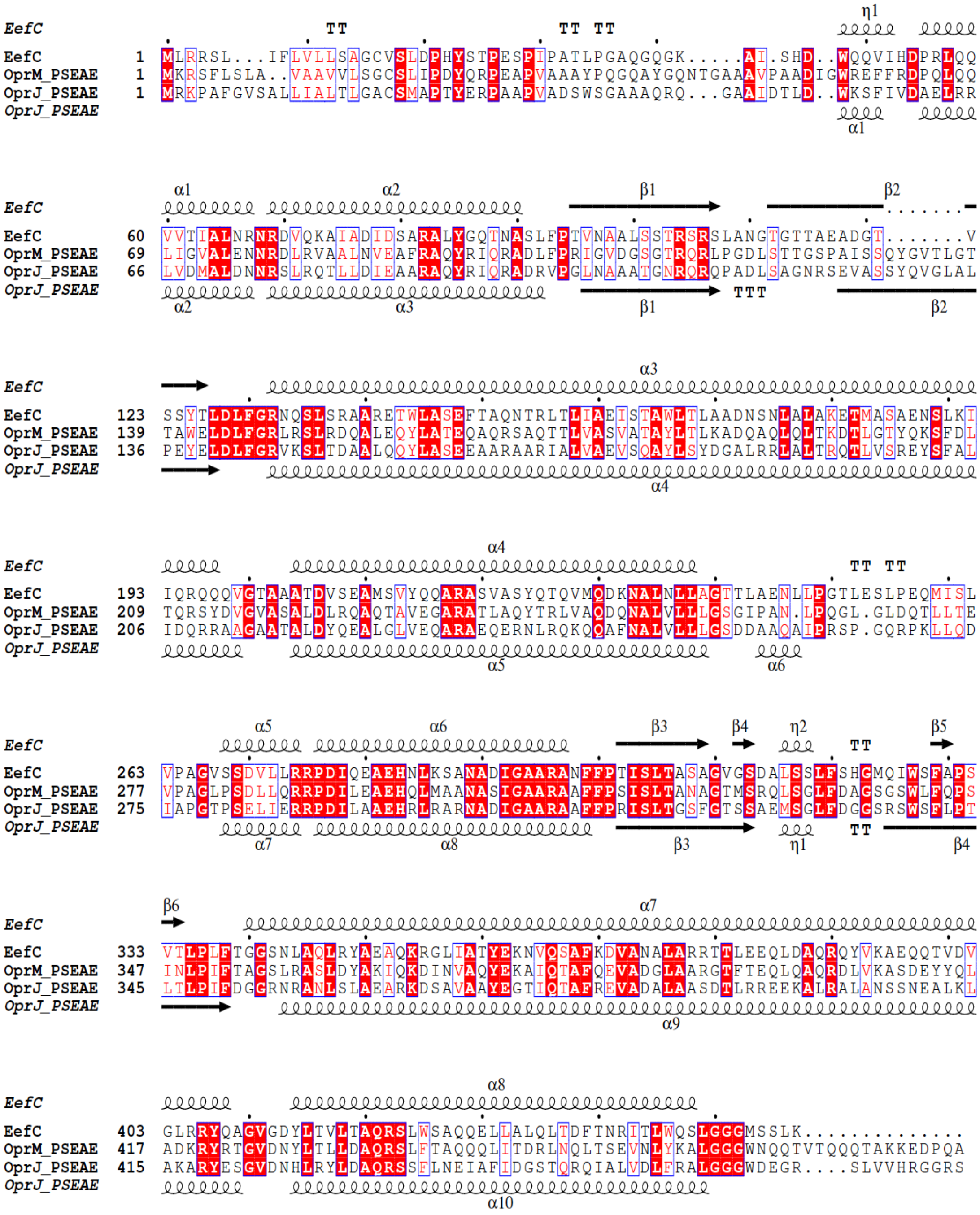

Figure S7. An MSA of EefC with closely related members of the OMF family. As can be seen, EefC is closely related to the OprJ/M/N branch of the OMF family, allowing for reliable homology modelling using them as templates

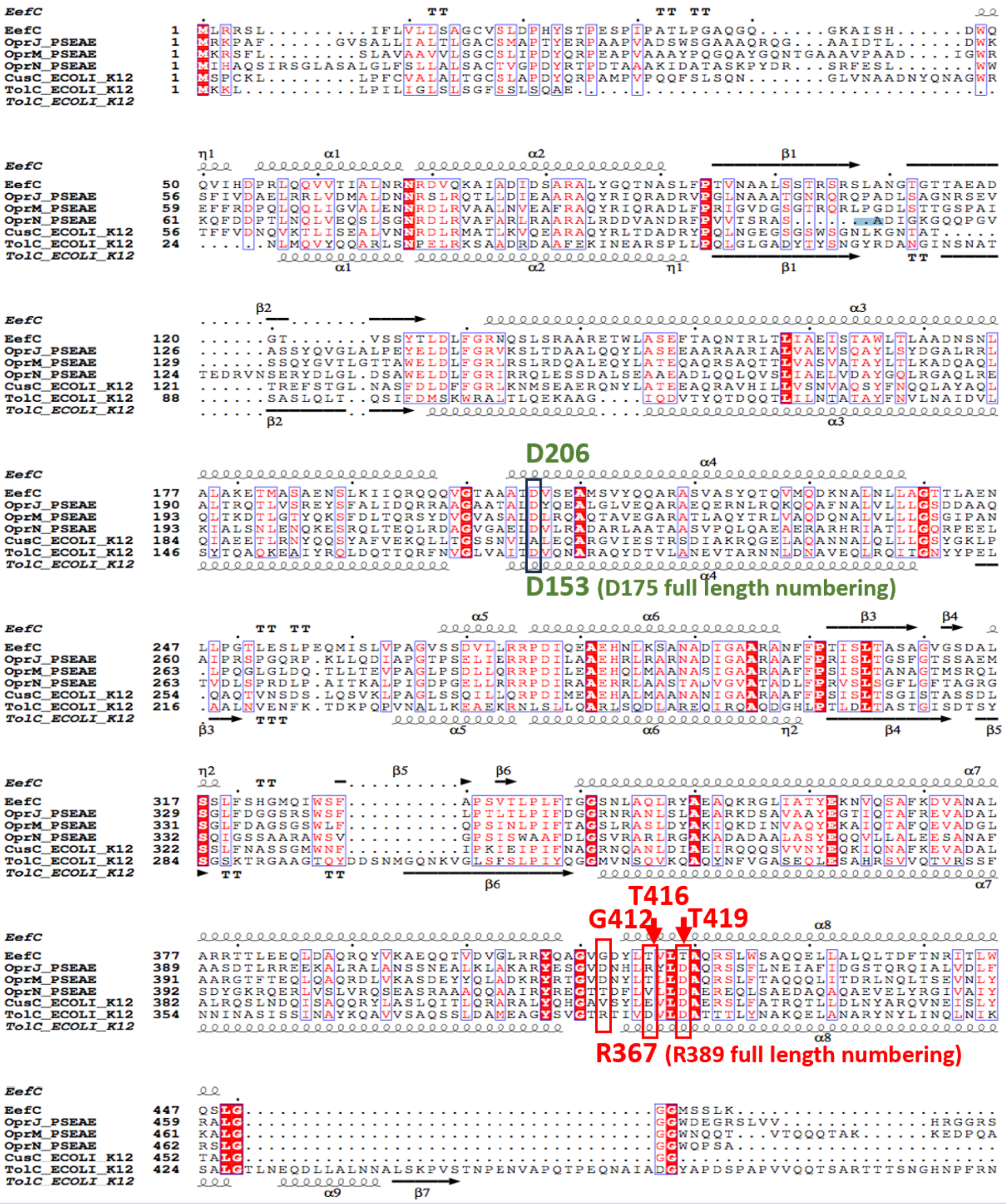

Figure S8. A wider MSA of OMFs, including TolC, showing the closer association of EefC to the OprJ/M/N clade of OMFs than to TolC, and highlighting some critical differences in gating residues controlling the opening of the EefC vs TolC channels. Residue numbers are given for full-length, non-processed EefC, while for historical reasons, both full-length and mature protein residues numbers (as per (Koronakis et al., 2000)) are provided for TolC.

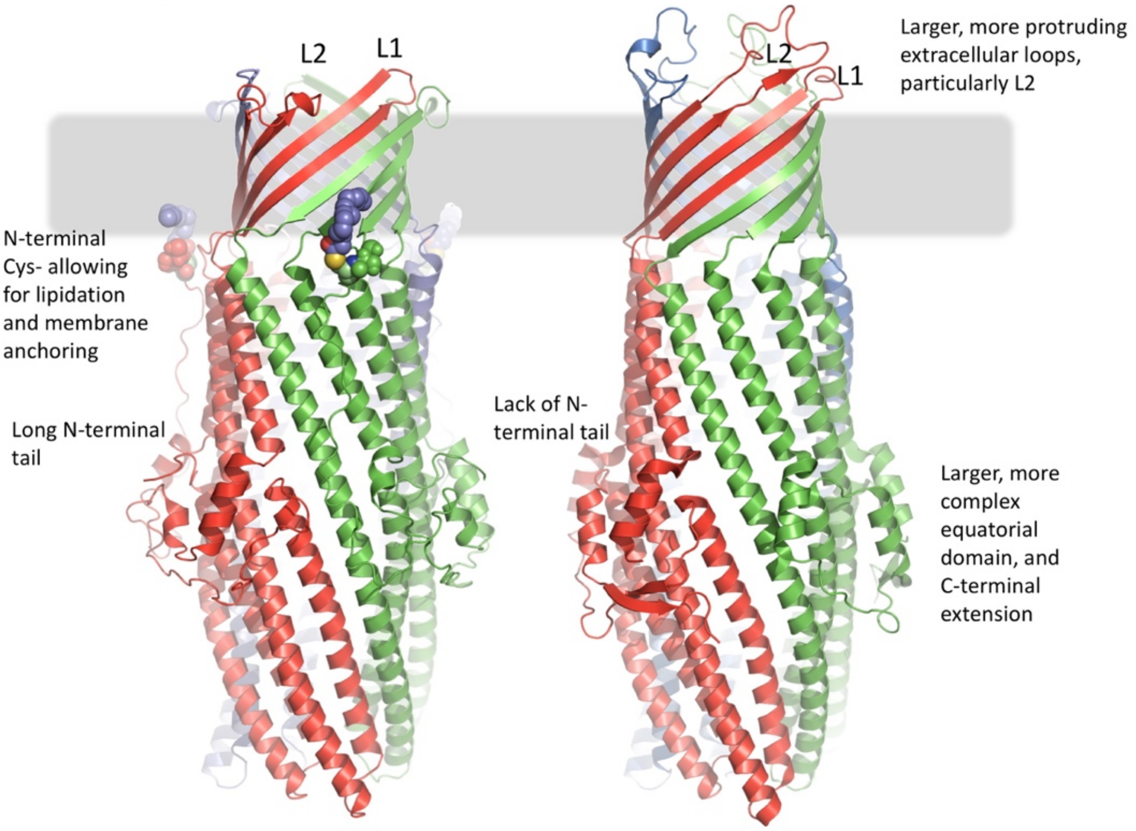

Figure S9. Key structural differences between EefC (left) and TolC channels as discussed in the main text.

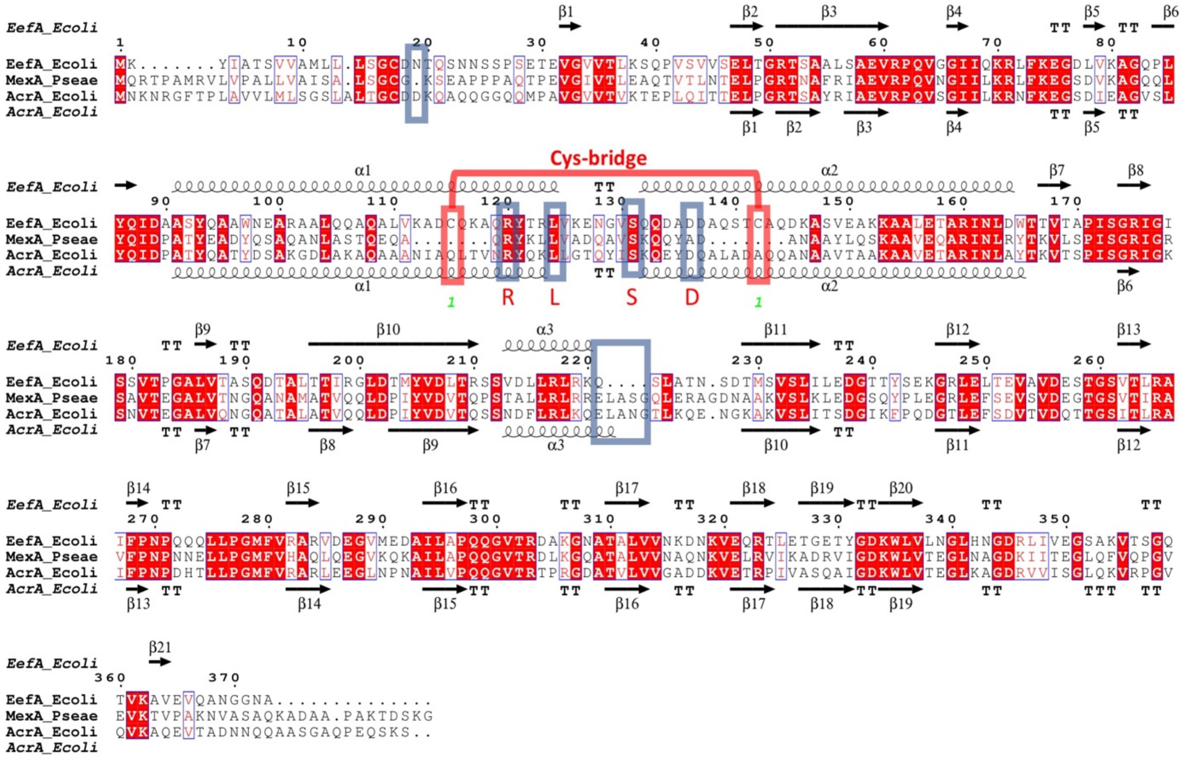

Figure S10. An MSA of EefA with periplasmic adapter proteins of known structure, used as templates for its structural modelling. Secondary structure elements are indicated above the and below the alignment for EefA (top) and AcrA, respectively. The RLS motif at the tip of the alpha-helical hairpin domain of the PAPs is highlighted with a set of boxes and letters, as is the 4-residue deletion in EefA relative to the other PAPs as discussed in the text.

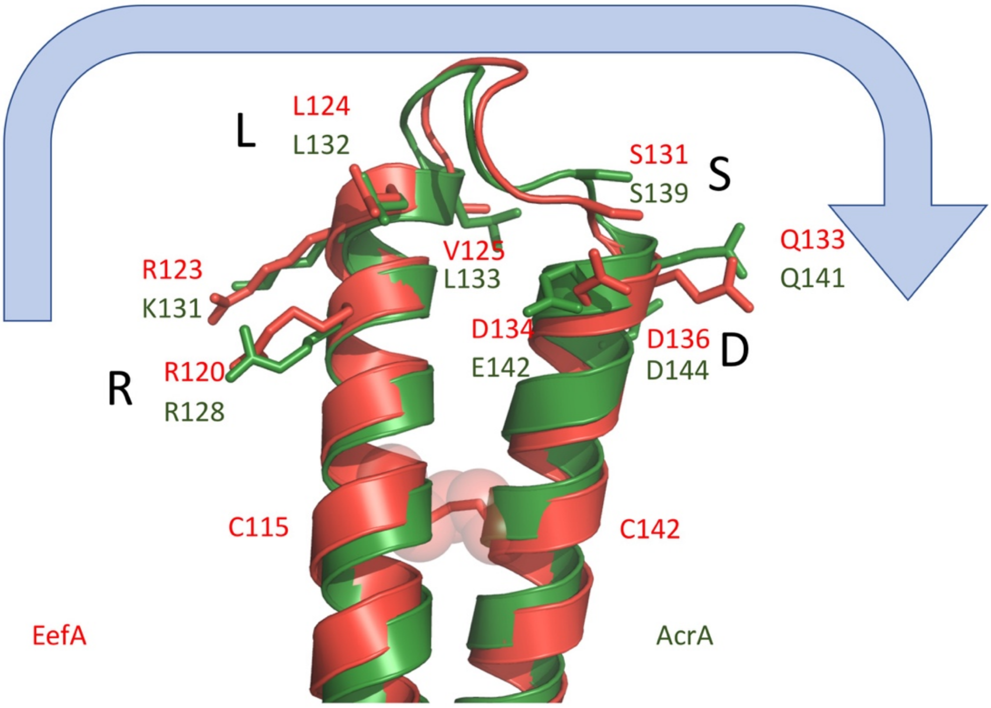

Figure S11. Residue conservation at the tip of the alpha-helical hairpin domain of EefA vs AcrA suggest radically different OMF-docking patterns, and likely cross-incompatibility between the PAPs and their respective OMFs.

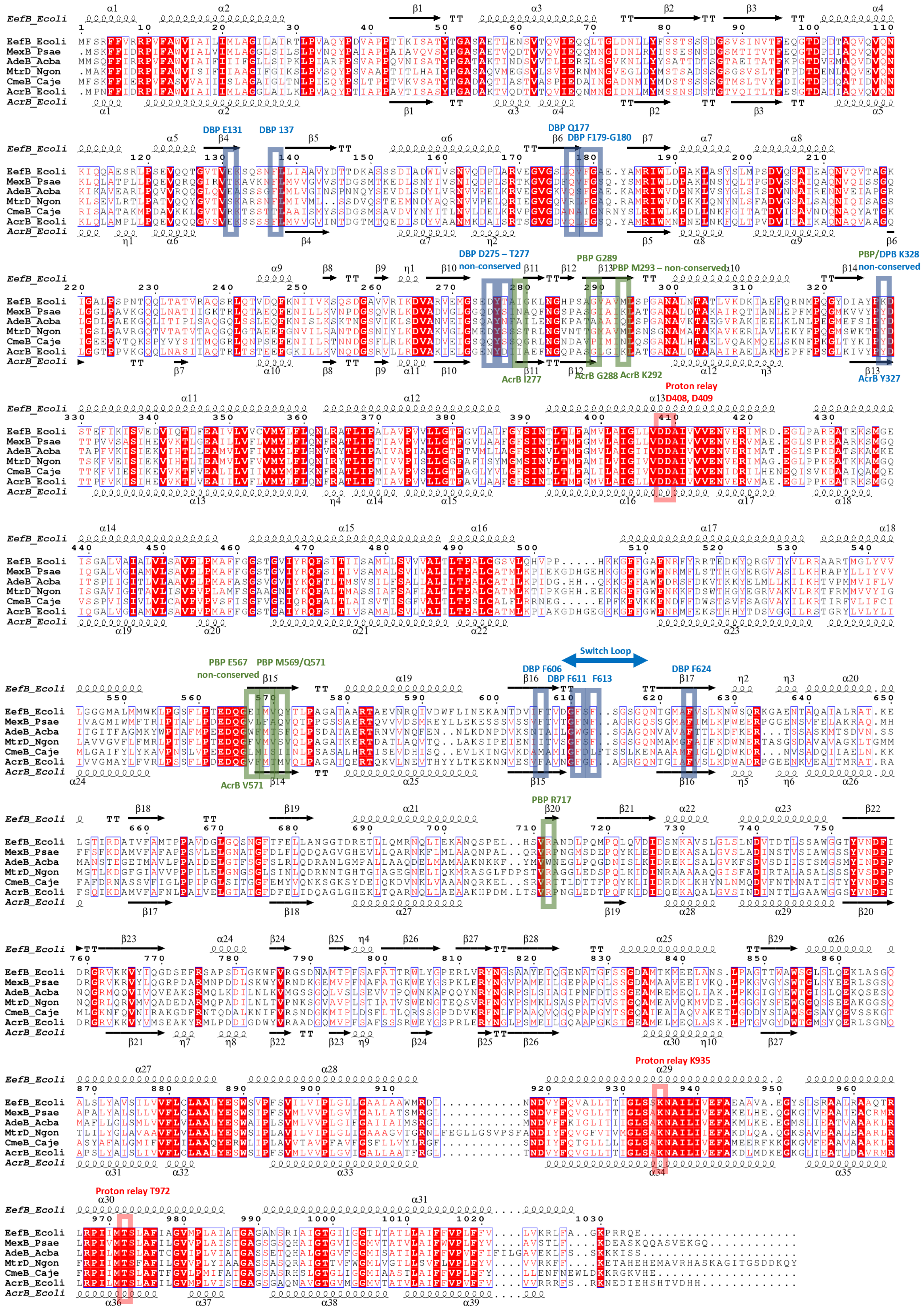

EefB_Ecoli
MexB_Psae
AdeB_Acba
MtrD_Ngon
CmeB_Caje
AcrB_Ecoli
DBP E131
DBP 137
DBP Q177
DBP F179-G180
DBP D275 – T277
non-conserved
PBP G289
PBP M293 – non-conserved
PBP/DPB K328
non-conserved
AcrB I277
AcrB G288
AcrB K292
AcrB Y327
Proton relay
D408, D409
PBP E567
non-conserved
PBP M569/Q571
AcrB V571
Switch Loop
DBP F606
DBP F611
F613
DBP F624
PBP R717
Proton relay K935
Proton relay T972

Figure S12. Structural alignment comparing the predicted organisation of EefB (top) with the empirical structure of *E. coli* AcrB (bottom) and sequences of other RND transporters of known structure.

Key residues associated with proton-coupling, as well as the residues lining the Proximal Binding Pocket (PBP) and Distal Binding Pocket (DBP), are highlighted using red, green and blue boxes respectively, and their numbers are shown on top of the alignment as per EefB full-length sequence. Where there is a major discrepancy in sequence between EefB and AcrB, the corresponding AcrB residues are also noted below the alignment.

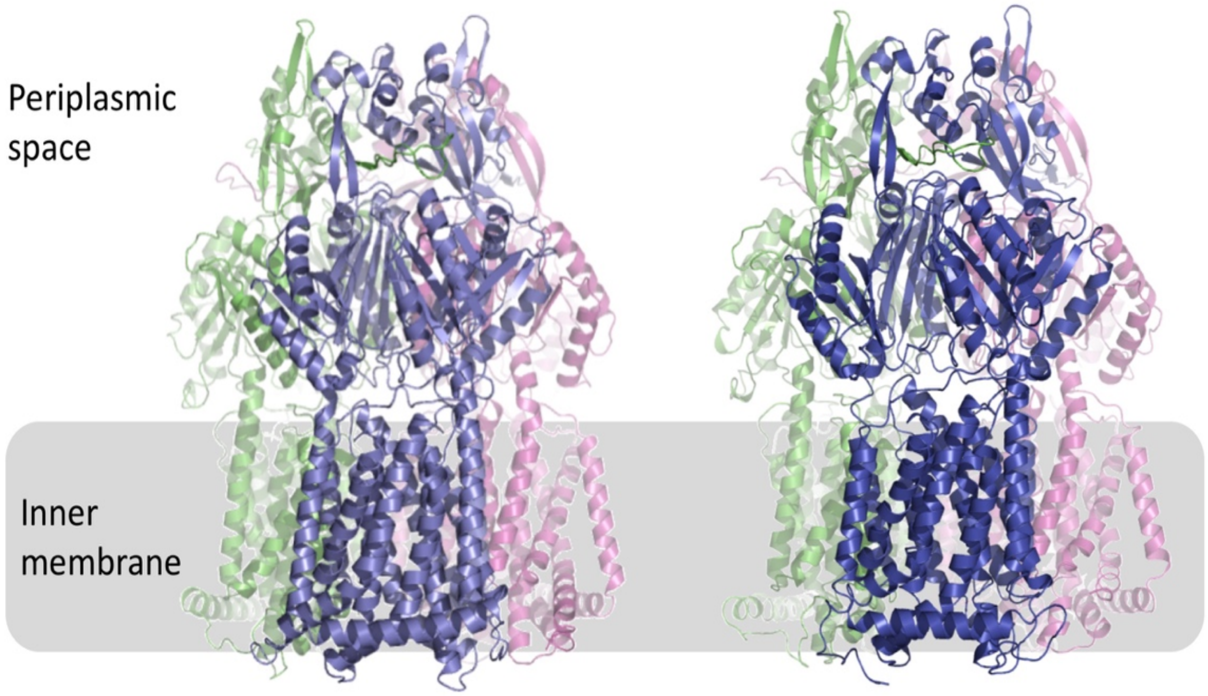

Figure S13. Comparison of the predicted structures of the assembled EefB trimer (left) and the experimental structure of AcrB (based on 2GIF.pdb) (Seeger et al., 2006).

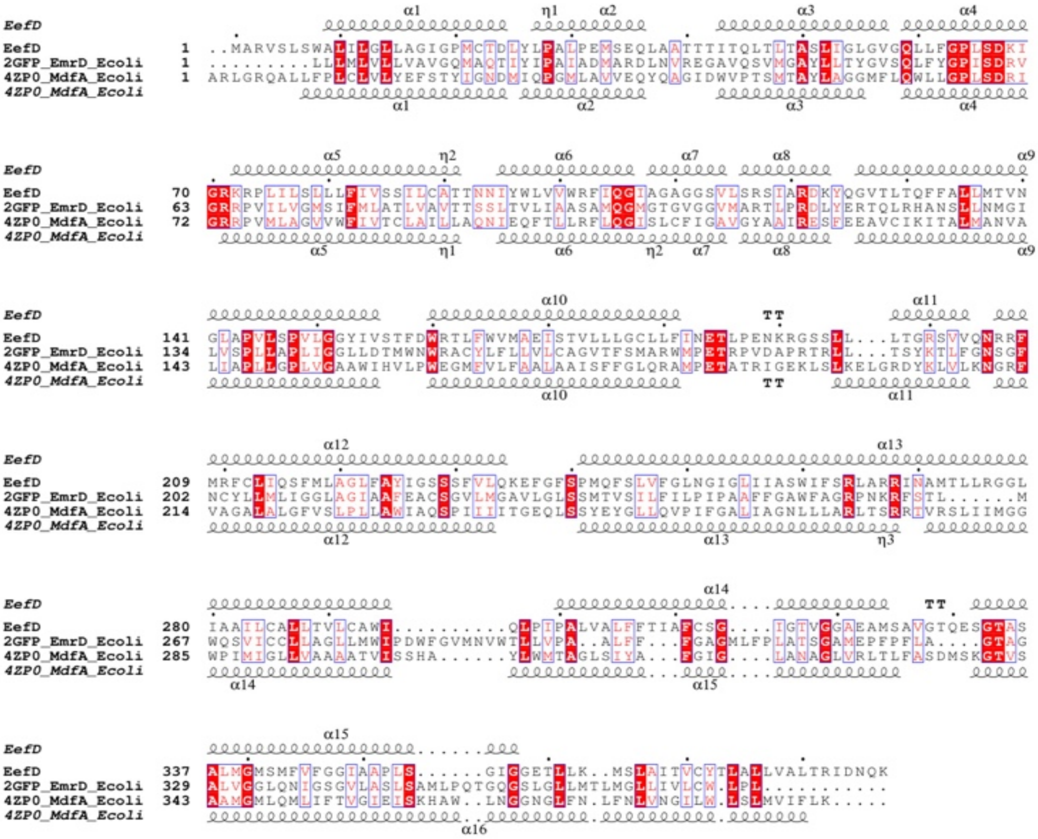

Figure S14. A MSA comparing the predicted structure of EefD with the empirically determined structures of *E. coli* EmrD and MdfA. Secondary structure elements are shown above and below the alignment.

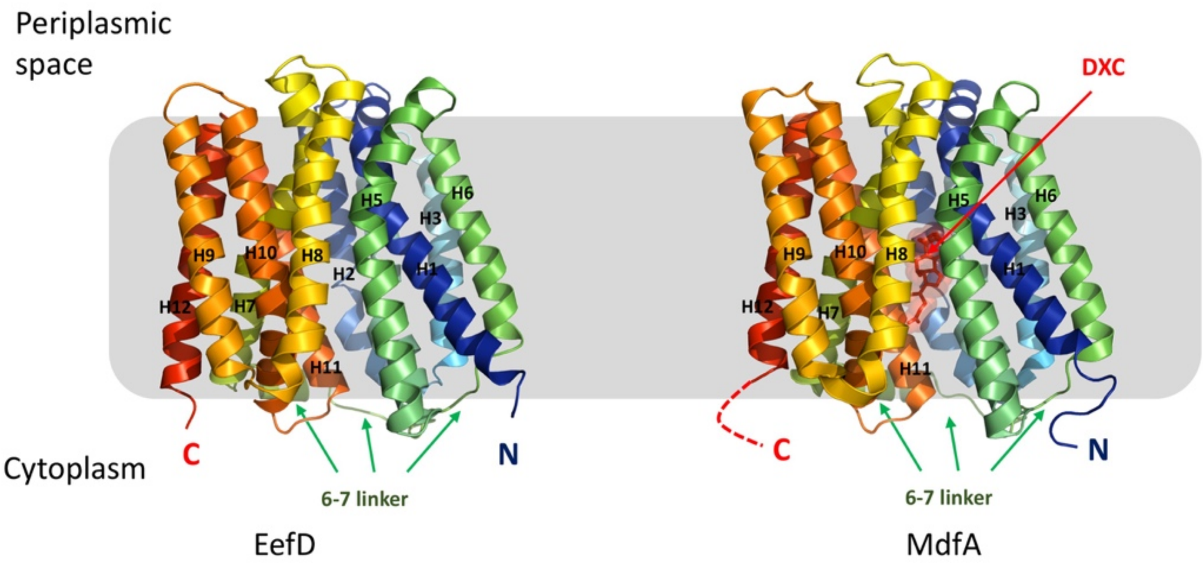

Figure S15. Side-by-side comparison of the predicted structure of EefD (left, modelled in an inward facing state) with the empirically determined structure of *E. coli* MdfA in complex with a deoxycholate ligand (DXC), as per 4ZP0.pdb (Heng et al., 2015).

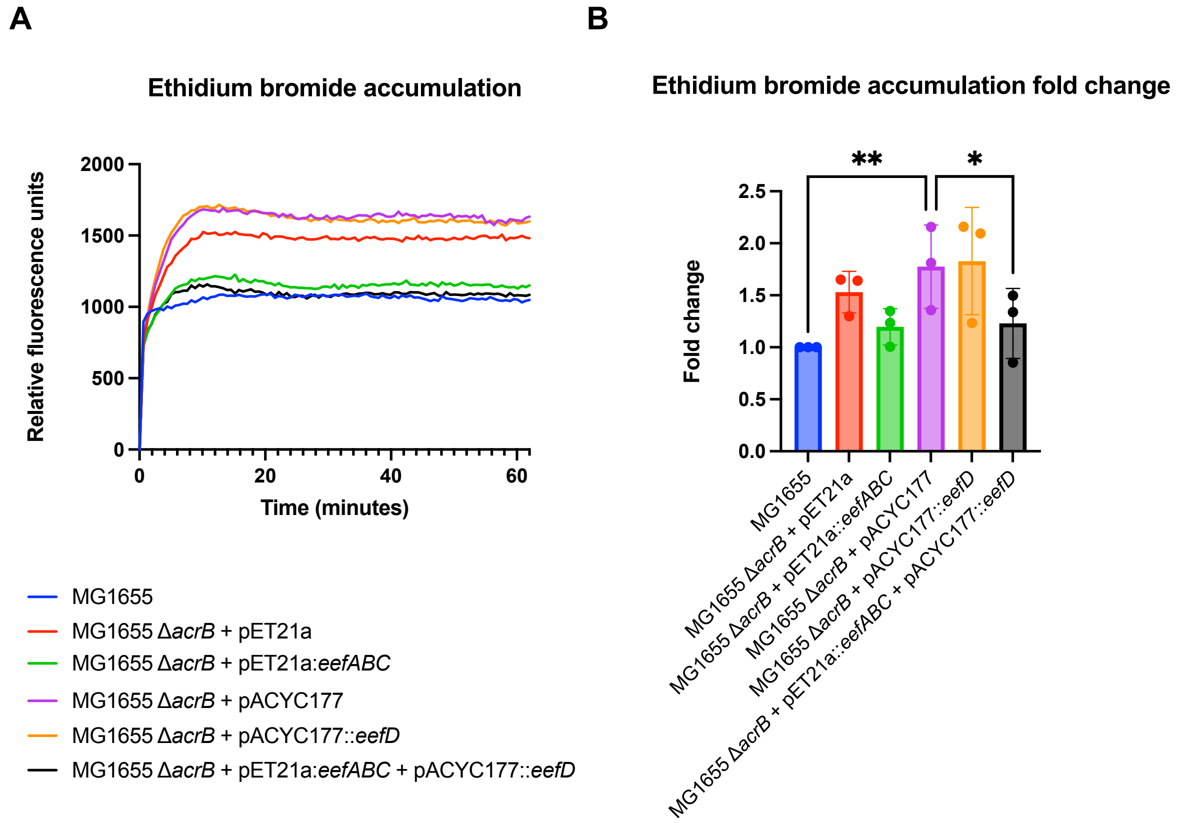

Figure S16. Ethidium bromide accumulation assay for gain of function *E. coli* strains. A) Accumulation of ethidium bromide over time. B) Endpoint fluorescence fold-change normalized to *E. coli* MG1655. Significance was determined one-way-Anova analysis, comparing each endpoint fluorescent value to that of the wildtype *E. coli* MG1655. Data is determined from three biological repeats with error bars showing standard deviation.

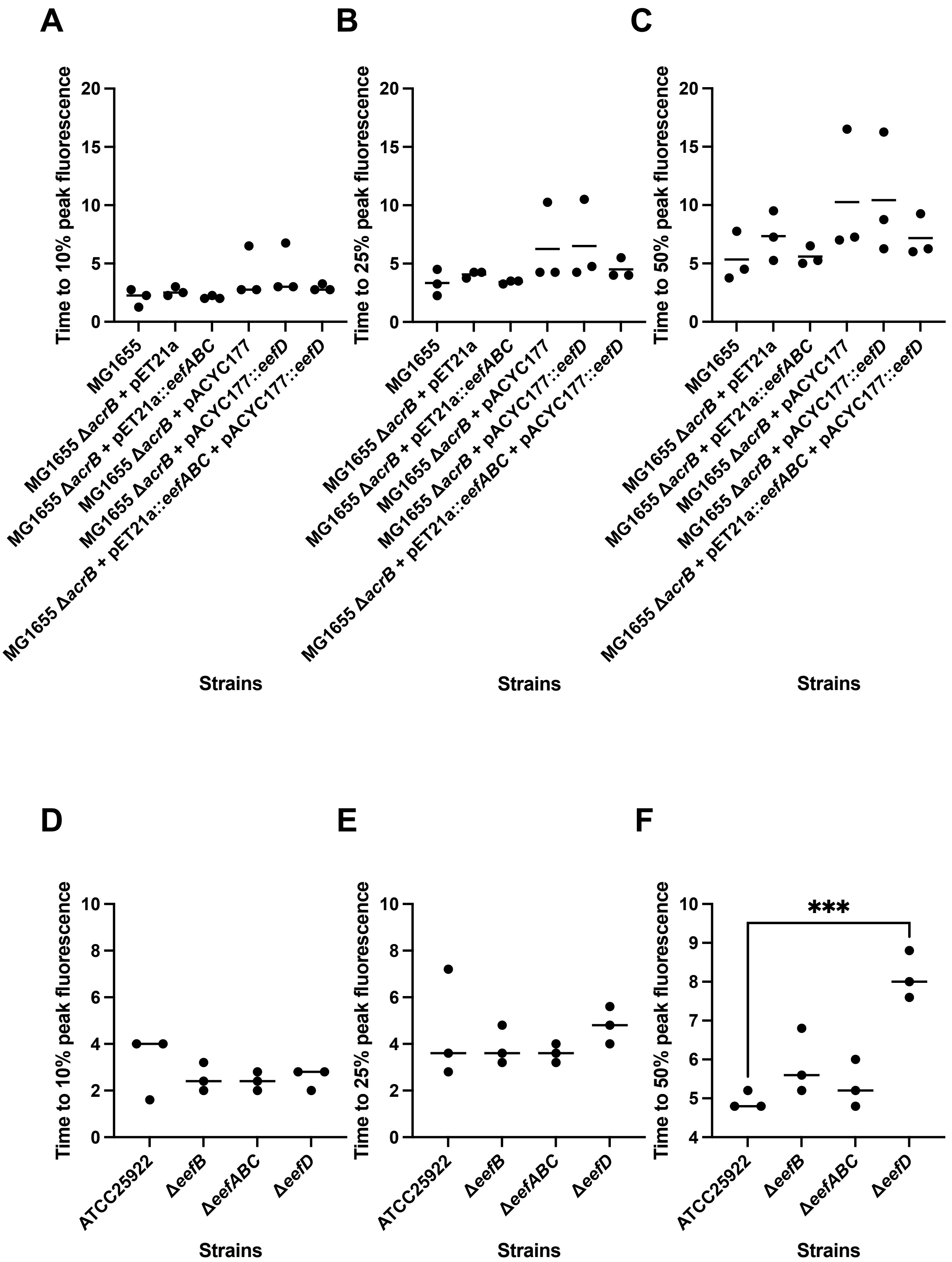

Figure S17. Ethidium Bromide efflux for gain and loss of function *E. coli* strains. Strains were preloaded with ethidium bromide in the presence of a proton motive force inhibitor before being reenergised with glucose. The time taken ethidium bromide

fluorescence within the cell to decrease by 10%, 25% and 50% respectively in a gain-of-function *E. coli* MG1655 background are shown in in panels A, B and C. Panels D, E, and F summarise the same experiment, time taken for fluorescence to drop by 10%, 25% and 50%, but for loss-of-function strains in the *E. coli* ATCC 25922 background. Time is shown in minutes. Level of significance was calculated using one-way-Anova analysis. Data are showing the mean from three biological repeats.

Structural analysis of the EefABC models.

EefC – structure:

Our initial sequence analysis revealed several intriguing and unexpected features of the EefABC pump. We first focused on EefC which, as expected, was revealed to be a TolC paralogue, and is a member of the outer membrane factor (OMF) family (Paulsen et al., 1997). Within the *E. coli* genome there have been only 4 OMF family members identified so far – namely *tolC*, *mdtP* (*yjcP*), *mdtQ* (*yohH*) and *cusC* (*ylcB*)(Sulavik et al., 2001). Sequence alignment of EefC with the endogenous *E. coli* OMFs revealed that it is relatively distant to the TolC branch, but closely aligned to the metal pump-associated CusC and MdtP/MdtQ branch of the family (See Fig. S6). MAFFT multiple sequence alignment and the corresponding phylogenetic tree revealed that EefC is most (see Fig. S6 and S7). CusC is produced in a single operon with the RND-metal pump CusBA and is implicated in resistance against Cu(I)/Ag(I) ions generated under anaerobic conditions (Franke et al., 2003).

Comparison with other OMFs of known structure, including the *Pseudomonas* OMF channels OprM ((Akama et al., 2004) (1WP1.pdb) ; (Monlezun et al., 2015) (4Y1K.pdb)), OprN (5AZO.pdb; 5AZP.pdb) and OprJ (5AZS.pdb) (Yonehara et al., 2016) , as well as the Salmonella ST50 (Guan et al., 2015) (5BUN.pdb) and VceC (1YC9.pdb (Federici et al., 2005) confirmed that the closest relatives within the wider OMF family appear to be the *Pseudomonas* proteins OprM and OprJ (see Fig. S7), followed by the *CusC*, and correspondingly the former were used as structural templates for homology modelling (see Fig. S7).

From this structural alignment it is immediately notable, that there are very few gaps in the alignment between OprM/J and Eef (Fig. S7), which is in stark contrast to the TolC (Fig. **S8**). That allows for almost direct mapping of the aligned sequences of EefC onto OprM/J. The only notable exceptions are the marked shift in the position of the beta-2 strand of the first beta hairpin, resulting in significant shortening of the extracellular loop1, and secondly, the rather shortened C-terminus, which is significantly truncated, especially comparing to the TolC-branch of the OMF family (Fig. S8). Side by side comparison of EefC vs TolC allows to identify the key differences between the two channels (Fig. S9).

Of particular note is that the primary gating loop sequences (located on the tips of H3/H4 and H7/H8 helical hairpins in TolC) are not conserved amongst the TolC

paralogues within *E. coli* (Fig. S8), which is consistent with their functioning with dedicated PAPs (and correspondingly transporter pairs) and as a result not being cross-reactive.

The comparison of these gating-loop regions in EefC (which correspond to the tips of the H3/H4 and H7/H8 helices in TolC) reveals that they are highly divergent, suggesting that their locking mechanism is markedly different in EefC from that observed in TolC, and hence EefC is unlikely to work with PAPs recognizing TolC "out of the box", but rather likely only interacting with its cognate PAP EefA.

This is particularly prominent on the tip of the H7/H8 hairpin, which harbours several key residues responsible for gating the TolC channel. While Y362 (TolC) is critically conserved across the family, the R367 (TolC), which links across to H4 binding to the conserved D153 (TolC; D206 in EefC) forming the so called "primary gate" thus sealing the TolC channel, (Andersen et al., 2002a, Bavro et al., 2008) is substituted in EefC by a small non-polar residue G412 making such interaction impossible.

Furthermore, the so-called "secondary gate", formed of the prominently conserved double aspartate ring (which in TolC if composed of D371/D374 from all three protomers (TolC 1EK9.pdb numbering)), and which forms the basis of ion-selectivity in TolC (Andersen et al., 2002b, Schulz and Kleinekathöfer, 2009) is fully absent and substituted by a small-hydroxylated residues instead – namely T416 and T419 (YLTVLTA), a feature unique to the EefC, as although D371 position is variable across the OMF family, the D374 position is remarkably conserved (even within the OprM/CusC clusters), and the only other exception are the rather deviant AatA proteins associated with dispersin secretion discussed above (VITYSSV). This changes the electrostatic properties of the channel dramatically, which is further exacerbated by the presence of the bulky L415 and L418, which provide a hydrophobic seal of the periplasmic end of the channel. The inner lining of the EefC tube is also markedly more hydrophilic, lined with a number of serine residues.

It is important to note that the residue corresponding to the EefC R422, is conserved across *Pseudomonas* OprJ/OprM/OprN clade, as well as in the CusC group of OMFs, while it is markedly different in TolC-branch of the family (including MdtP and MdtQ). In the former group of OMFs, its role seems to be coordination of the upper-ring of Asp-residues forming the "secondary gate" electrostatic seal, however in EefC the residue corresponding to the second Asp is T419, and such an interaction is

impossible. Taken together, the significant differences in the EefC gating mechanism may contribute to the observed differential ion and substrate selectivity of the pump.

These are not the only similarities with the OprM/N/J family however. The EefC exhibits long-N-terminal tail, which is some 30 residues longer than the corresponding region of TolC, but very much in line with the length seen in OprN/J. Furthermore, the N-terminus of EefC contains a cysteine residue, which isn't present at all in the TolC branch of the family, but which is conserved in the OprM/CusC (Monlezun et al., 2015), where it is a target to lipidation, usually taking the form of palmitoylation, which facilitates its insertion into the outer membrane. Indeed, as seen in Fig. S9 we used the OprM structure 4Y1K.pdb (Monlezun et al., 2015) to model the palmitoylation of the C-terminal cysteine.

Moving to the outer-membrane-inserted β-barrel domain, in comparison to the TolC, EefC features markedly shorter extracellular loops, in particular the L2 loop, which occludes TolC opening (Vaccaro et al., 2008); suggesting more-open state of the EefC channel. Such OMP loops are also prominent targets of protective antibodies (Domínguez-Medina et al., 2020), and the non-protruding loops can serve to avoid antibody restriction and LPS occlusion.

Finally, there are significant differences in the organisation of the equatorial domain, which in EefC is composed of the residue ranges P31-R69 (N-terminal tail and alpha-helix H2); G240-P276 (the long loop connecting the hairpin-forming helices H4 and H6, which includes a short helix H5); plus a very short C-terminal tail (G450-K457) that follows the H8 helix. The corresponding regions in TolC are again markedly different, with the N-terminal section contributing to the equatorial domain being much shorter (S20-P37 full-length TolC numbering); the central section being also slightly shorter (G209-P232), while the C-terminal tail being dramatically longer at over 60 residues (G427-N493). The features of EefC equatorial domain match nearly perfectly the OprJ/OprN leaving virtually no gap in the alignment.

EefA – structure:

Our attention then turned to the PAP protein in the EefABC system, EefA which was also analysed in a similar way, revealing a sequence identity of 53.85% to the *Pseudomonas* MexA; but only 50.13% against the *E.coli* AcrA. Due to the availability of more complete full-length experimental templates AcrA homology models were created both using MexA (2v4d.pdb; (Symmons et al., 2009)) and AcrA (5v5s.pdb;

(Wang et al., 2017)) as a template (utilising both I-TASSER and Swiss-model tools) (Fig. S10). While neither of the models got to the same level of confidence as EefC, the alignments with the known PAP structures are readily interpretable allowing unambiguous interpretation of the protein features.

Alignment of EefA and AcrA results in a direct amino-acid match with only 2 gaps in the alignment, one in the unstructured N-terminal tail, and another at position Q221 (EefA), which has a 4-residue-long deletion relative to AcrA. This region corresponds to the C-terminal end of alpha-helix 3 that is flanking the beta-barrel domain in PAPs, and it is also notable that shortening the same position is observed in MdtA/MdsA branch of the family, however it is not predicted to affect RND-binding (McNeil et al., 2019).

Despite overall similarity with MexA/AcrA, there are distinctive differences in the organisation of the EefA, notably in its α-hairpin domain (Fig. S11). While the RLS motif, which is speculated to be involved in PAP-OMF interaction (Kim et al., 2010, Song et al., 2014) appears to be preserved: R120/L124/S131/D136 (vs R128/L132/S139/D144 in *E.coli*), there are a number of significant changes in the adjacent residues, notably R123 (K131); V125 (L133); D134 (E142), which are predicted to produce steric clashes that would preclude its direct compatibility with TolC, and hence, such residues are likely to be playing a discriminatory role engaging with EefC.

Another unique feature is the predicted close proximity of C115 and C142 in the α-hairpin domain, the fold of which create a plausible condition for the formation of a Cys-Cys bridge. While the role of such a bridge is difficult to predict, it is very distinctive and not observed in any other known PAPs.

<u>EefB - structure:</u>

The RND-transporter component of the EefABC is the EefB, a large integral membrane protein which is predicted to form a functional trimer similar to other transporters in the family. Alignment of the EefB with other RND transporters of known function, including AcrB (*E. coli*); MtrD (*N. gonorrhoeae*); MexB (*P. aeruginosa*); CusB (*C. jejuni*) and AdeB (*A. baumannii*) revealed that they are highly similar, with CusB and MtrD being most divergent, while AcrB and MexB being equidistant to EefB (see Fig. S12).

This close similarity (against MexB 56.8% identity in 1038 residues overlap; Score: 2995.0; Gap frequency: 0.5%; against AcrB 57.0% identity in 1033 residues overlap; Score: 2997.0; Gap frequency: 0.7% - fewer gaps in MexB) allowed to generate high-fidelity homology model of EefB (I-TASSER C-score 0.89; range -5,2) using MexB as a template, which was chosen due to the fewer gaps in the alignment (0.7% vs 0.5% for AcrB) and slightly higher residue overlap with EefB (1038 vs 1033 residue overlap respectively).

Overall, the alignment of with MexB (and AcrB) produces very few gaps (the longest is 2 residues long) allowing for unequivocal attribution of secondary structure elements (Fig. S12). As can be seen from the side-by-side comparison of the EefB and AcrB, both present a virtually identical architecture (Fig. S13) and an estimated RMSD of around 1.8 Å over the C-alpha backbone, with only notable differences being the shortened loop connecting TM helices α16 and α17 in EefB (residues 498-507); and the slightly shorter C-terminal tail. Consistent with this, the critical proton-relay residues found in MexB D407, D408, K939 and T976 (Guan and Nakae, 2001)(corresponding to D407, D408, K940 and T978 of AcrB); (Su et al., 2006); (Takatsuka and Nikaido, 2006) are preserved in EefD (D408, D409, K935 and T972 respectively).

While a full analysis of the drug binding pockets and efflux ducts of EefB is beyond the remit of the current manuscript, the Access and Deep pockets (AP and DP) (Nakashima et al., 2013, Ramaswamy et al., 2018) conservation suggests a closer relation to the MexB/AcrB rather than to MexY type transporters, with notable preservation of the key hydrophobic residues in the so-called phenylalanine cluster [numbering as per MexB, with EefB numbering in brackets]: F136 (F137); F178 (F179); G179 (G180); F610 (F606); F615 (F611); F617 (F613 – switch loop); F628 (F624) (Ramaswamy et al., 2018).

<u>EefD – structure</u>:

The last member of the operon is EefD, and our analysis indicated that this is a secondary transporter that belongs to the 2.A.1.2 group of Transporter Classification Database (TCDB) which groups MFS (<u>M</u>ajor <u>F</u>acilitator <u>S</u>uper-)family transporter, related to MDR-function including the Bcr/CflA, EmrD and MdfA branches (Reddy et al., 2012) (Quistgaard et al., 2016). This resulted in high-confidence homology models,

revealing a classic 6+6 TM-helical arrangement which is closely related to the general topology observed in MdfA (Adler and Bibi, 2002) (Fig. S14).

Comparison of the homology model of EefD with the available structures of EmrD (Yin et al., 2006) and MdfA (Heng et al., 2015, Nagarathinam et al., 2018) further confirmed that the EefD is more closely related to the latter (Fig. S15), however there are significant differences in the substrate binding cavity.

Notably, the conserved acidic residue D34 (MdfA) involved in protonation and substrate coupling cycle of the transporter is preserved, while the second acidic residue E26(MdfA), is instead substituted by an aliphatic A16, suggesting a difference in the cycle. Furthermore, the residue Y127 (MdfA) which makes contact with D34 in the outwardly-open state of the transporter (Nagarathinam et al., 2018) is substituted by a positively charged R117, making such interaction impossible.

Additional analysis reveals significant conservation of the so-called motif A, and the critical residue E132 (D122 in EefD) that anchors TM4 to it, however the interactions that are anchoring the motif A to the linker helix 6-7 (notably MdfA residues R198 and D211) are not conserved. Finally there are a number of changes along the central substrate-binding cavity, suggesting significant divergence of potential substrates between EefD and MdfA (see table below for details). These discrepancies make predictions of the possible substrates of the EefD problematic, although some overlap with MdfA can be expected (Lewinson et al., 2003) e.g. chloramphenicol/thiamphenicol and lipophilic cations such as ethidium.

Overall, our analysis of the EefABC reveals unexpected similarities to the tripartite pumps OprM-MexAB and OprJ-MexCD in *Pseudomonas* (Masuda et al., 2000), rather than to any endogenous *E.coli* paralogues, which may suggest that the precursors of these pumps have been acquired via a lateral gene transfer event, similar to some other plasmid encoded efflux/secretion systems (e.g. the EAEC virulence plasmid pAA2) (Imuta et al., 2008) and correspondingly may play a role in virulence of the *E.coli*.

References

ADLER, J. & BIBI, E. 2002. Membrane topology of the multidrug transporter MdfA: Complementary gene fusion studies reveal a nonessential C-terminal domain. *Journal of Bacteriology,* 184**,** 3313-3320.

AKAMA, H., KANEMAKI, M., YOSHIMURA, M., TSUKIHARA, T., KASHIWAGI, T., YONEYAMA, H., NARITA, S. I., NAKAGAWA, A. & NAKAE, T. 2004. Crystal structure of the drug discharge outer membrane protein, OprM, of Pseudomonas aeruginosa: Dual modes of membrane anchoring and occluded cavity end. *Journal of Biological Chemistry,* 279**,** 52816-52819.

ANDERSEN, C., KORONAKIS, E., BOKMA, E., ESWARAN, J., HUMPHREYS, D., HUGHES, C. & KORONAKIS, V. 2002a. Transition to the open state of the TolC periplasmic tunnel entrance. *Proceedings of the National Academy of Sciences of the United States of America,* 99**,** 11103-11108.

ANDERSEN, C., KORONAKIS, E., HUGHES, C. & KORONAKIS, V. 2002b. An aspartate ring at the TolC tunnel entrance determines ion selectivity and presents a target for blocking by large cations. *Molecular Microbiology,* 44**,** 1131-1139.

BAVRO, V. N., PIETRAS, Z., FURNHAM, N., PÉREZ-CANO, L., FERNÁNDEZ-RECIO, J., PEI, X. Y., MISRA, R. & LUISI, B. 2008. Assembly and Channel Opening in a Bacterial Drug Efflux Machine. *Molecular Cell,* 30**,** 114-121.

DOMÍNGUEZ-MEDINA, C. C., PÉREZ-TOLEDO, M., SCHAGER, A. E., MARSHALL, J. L., COOK, C. N., BOBAT, S., HWANG, H., CHUN, B. J., LOGAN, E., BRYANT, J. A., CHANNELL, W. M., MORRIS, F. C., JOSSI, S. E., ALSHAYEA, A., ROSSITER, A. E., BARROW, P. A., HORSNELL, W. G., MACLENNAN, C. A., HENDERSON, I. R., LAKEY, J. H., GUMBART, J. C., LÓPEZ-MACÍAS, C., BAVRO, V. N. & CUNNINGHAM, A. F. 2020. Outer membrane protein size and LPS O-antigen define protective antibody targeting to the Salmonella surface. *Nature Communications,* 11.

FEDERICI, L., DU, D., WALAS, F., MATSUMURA, H., FERNANDEZ-RECIO, J., MCKEEGAN, K. S., BORGES-WALMSLEY, M. I., LUISI, B. F. & WALMSLEY, A. R. 2005. The crystal structure of the outer membrane protein VceC from the bacterial pathogen Vibrio cholerae at 1.8 Å resolution. *Journal of Biological Chemistry,* 280**,** 15307-15314.

FRANKE, S., GRASS, G., RENSING, C. & NIES, D. H. 2003. Molecular analysis of the copper-transporting efflux system CusCFBA of Escherichia coli. *Journal of Bacteriology,* 185**,** 3804-3812.

GUAN, H. H., YOSHIMURA, M., CHUANKHAYAN, P., LIN, C. C., CHEN, N. C., YANG, M. C., ISMAIL, A., FUN, H. K. & CHEN, C. J. 2015. Crystal structure of an antigenic outer-membrane protein from Salmonella Typhi suggests a potential antigenic loop and an efflux mechanism. *Scientific Reports,* 5.

GUAN, L. & NAKAE, T. 2001. Identification of essential charged residues in transmembrane segments of the multidrug transporter MexB of Pseudomonas aeruginosa. *Journal of Bacteriology,* 183**,** 1734-1739.

HENG, J., ZHAO, Y., LIU, M., LIU, Y., FAN, J., WANG, X., ZHAO, Y. & ZHANG, X. C. 2015. Substrate-bound structure of the *E. coli* multidrug resistance transporter MdfA. *Cell Res,* 25**,** 1060-73.

IMUTA, N., NISHI, J., TOKUDA, K., FUJIYAMA, R., MANAGO, K., IWASHITA, M., SARANTUYA, J. & KAWANO, Y. 2008. The Escherichia coli efflux pump TolC promotes aggregation of enteroaggregative *E. coli* 042. *Infection and Immunity,* 76**,** 1247-1256.

KIM, H. M., XU, Y., LEE, M., PIAO, S., SIM, S. H., HA, N. C. & LEE, K. 2010. Functional relationships between the AcrA hairpin tip region and the TolC aperture tip region for the formation of the bacterial tripartite efflux pump AcrAB-TolC. *Journal of Bacteriology,* 192**,** 4498-4503.

KORONAKIS, V., SHARFF, A., KORONAKIS, E., LUISI, B. & HUGHES, C. 2000. Crystal structure of the bacterial membrane protein TolC central to multidrug efflux and protein export. *Nature,* 405**,** 914-9.

LEWINSON, O., ADLER, J., POELARENDS, G. J., MAZURKIEWICZ, P., DRIESSEN, A. J. M. & BIBI, E. 2003. The Escherichia coli multidrug transporter MdfA catalyzes both electrogenic and electroneutral transport reactions. *Proceedings of the National Academy of Sciences of the United States of America,* 100**,** 1667-1672.

MASI, M., PAGES, J. M., VILLARD, C. & PRADEL, E. 2005. The eefABC multidrug efflux pump operon is repressed by H-NS in Enterobacter aerogenes. *J Bacteriol,* 187**,** 3894-7.

MASUDA, N., SAKAGAWA, E., OHYA, S., GOTOH, N., TSUJIMOTO, H. & NISHINO, T. 2000. Substrate specificities of MexAB-OprM, MexCD-OprJ, and MexXY-OprM efflux pumps in Pseudomonas aeruginosa. *Antimicrobial Agents and Chemotherapy,* 44**,** 3322-3327.

MCNEIL, H. E., ALAV, I., TORRES, R. C., ROSSITER, A. E., LAYCOCK, E., LEGOOD, S., KAUR, I., DAVIES, M., WAND, M., WEBBER, M. A., BAVRO, V. N. & BLAIR, J. M. A. 2019. Identification of binding residues between periplasmic adapter protein (PAP) and RND efflux pumps explains PAP-pump promiscuity and roles in antimicrobial resistance. *PLoS Pathog,* 15**,** e1008101.

MONLEZUN, L., PHAN, G., BENABDELHAK, H., LASCOMBE, M. B., ENGUÉNÉ, V. Y. N., PICARD, M. & BROUTIN, I. 2015. New OprM structure highlighting the nature of the N-terminal anchor. *Frontiers in Microbiology,* 6.

NAGARATHINAM, K., NAKADA-NAKURA, Y., PARTHIER, C., TERADA, T., JUGE, N., JAENECKE, F., LIU, K., HOTTA, Y., MIYAJI, T., OMOTE, H., IWATA, S., NOMURA, N., STUBBS, M. T. & TANABE, M. 2018. Outward open conformation of a Major Facilitator Superfamily multidrug/H+ antiporter provides insights into switching mechanism. *Nature Communications,* 9.

NAKASHIMA, R., SAKURAI, K., YAMASAKI, S., HAYASHI, K., NAGATA, C., HOSHINO, K., ONODERA, Y., NISHINO, K. & YAMAGUCHI, A. 2013. Structural basis for the inhibition of bacterial multidrug exporters. *Nature,* 500**,** 102-106.

PAULSEN, I. T., PARK, J. H., CHOI, P. S. & SAIER, M. H. 1997. A family of Gram-negative bacterial outer membrane factors that function in the export of proteins, carbohydrates, drugs and heavy metals from Gram-negative bacteria. *FEMS Microbiology Letters,* 156**,** 1-8.

QUISTGAARD, E. M., LOW, C., GUETTOU, F. & NORDLUND, P. 2016. Understanding transport by the major facilitator superfamily (MFS): structures pave the way. *Nat Rev Mol Cell Biol,* 17**,** 123-32.

RAMASWAMY, V. K., VARGIU, A. V., MALLOCI, G., DREIER, J. & RUGGERONE, P. 2018. Molecular determinants of the promiscuity of MexB and MexY multidrug transporters of Pseudomonas aeruginosa. *Frontiers in Microbiology,* 9.

REDDY, V. S., SHLYKOV, M. A., CASTILLO, R., SUN, E. I. & SAIER, M. H. 2012. The major facilitator superfamily (MFS) revisited. *FEBS Journal,* 279**,** 2022-2035.

SCHULZ, R. & KLEINEKATHÖFER, U. 2009. Transitions between closed and open conformations of TolC: The effects of ions in simulations. *Biophysical Journal,* 96**,** 3116-3125.
SEEGER, M. A., SCHIEFNER, A., EICHER, T., VERREY, F., DIEDERICHS, K. & POS, K. M. 2006. Structural asymmetry of AcrB trimer suggests a peristaltic pump mechanism. *Science,* 313**,** 1295-8.
SONG, S., HWANG, S., LEE, S., HA, N. C. & LEE, K. 2014. Interaction mediated by the putative tip regions of MdsA and MdsC in the formation of a Salmonella-specific tripartite efflux pump. *PLoS ONE,* 9.
SU, C. C., LI, M., GU, R., TAKATSUKA, Y., MCDERMOTT, G., NIKAIDO, H. & YU, E. W. 2006. Conformation of the AcrB multidrug efflux pump in mutants of the putative proton relay pathway. *Journal of Bacteriology,* 188**,** 7290-7296.
SULAVIK, M. C., HOUSEWEART, C., CRAMER, C., JIWANI, N., MURGOLO, N., GREENE, J., DIDOMENICO, B., SHAW, K. J., MILLER, G. H., HARE, R. & SHIMER, G. 2001. Antibiotic Susceptibility Profiles of Escherichia coli Strains Lacking Multidrug Efflux Pump Genes. *Antimicrobial Agents and Chemotherapy,* 45**,** 1126-1136.
SYMMONS, M. F., BOKMA, E., KORONAKIS, E., HUGHES, C. & KORONAKIS, V. 2009. The assembled structure of a complete tripartite bacterial multidrug efflux pump. *Proc Natl Acad Sci U S A,* 106**,** 7173-8.
TAKATSUKA, Y. & NIKAIDO, H. 2006. Threonine-978 in the transmembrane segment of the multidrug efflux pump AcrB of Escherichia coli is crucial for drug transport as a probable component of the proton relay network. *Journal of Bacteriology,* 188**,** 7284-7289.
VACCARO, L., SCOTT, K. A. & SANSOM, M. S. P. 2008. Gating at both ends and breathing in the middle: Conformational dynamics of TolC. *Biophysical Journal,* 95**,** 5681-5691.
WANG, Z., FAN, G., HRYC, C. F., BLAZA, J. N., SERYSHEVA, I. I., SCHMID, M. F., CHIU, W., LUISI, B. F. & DU, D. 2017. An allosteric transport mechanism for the AcrAB-TolC multidrug efflux pump. *eLife,* 6.
YIN, Y., HE, X., SZEWCZYK, P., NGUYEN, T. & CHANG, G. 2006. Structure of the multidrug transporter EmrD from Escherichia coli. *Science,* 312**,** 741-744.
YONEHARA, R., YAMASHITA, E. & NAKAGAWA, A. 2016. Crystal structures of OprN and OprJ, outer membrane factors of multidrug tripartite efflux pumps of Pseudomonas aeruginosa. *Proteins: Structure, Function and Bioinformatics,* 84**,** 759-769.
